# Supplementary material for: Proximity-Induced Rewiring of Oncogenic Kinase Triggers Apoptosis
Source: ACS Cent Sci. 2026 Apr 9;12(4):473–83. doi: 10.1021/acscentsci.5c02202 (PMC13107213; doi:10.1021/acscentsci.5c02202)
Supplement: Supplementary file 2 [file oc5c02202_si_002.pdf]

Supporting Information: Chemical Synthesis Methods

Proximity-induced rewiring of oncogenic kinase triggers apoptosis

Manuel L. Merz[1,¶], Veronika M. Shoba[1,¶], Rajaiah Pergu[1,2,3,¶], Zachary C. Severance[1,¶], Dhanushka N. P. Munkanatta Godage[1], Arghya Deb[1], Hui Si Kwok[4,5], Prashant Singh[1], Sameek Singh[1], Jonathan B. Allen[9], Wenzhi Tian[1], Pallavi M. Gosavi[4,5], Santosh K. Chaudhary[1], Viktoriya Anokhina[1], Ellen L. Weisberg[6], N. Connor Payne[4,7,8], Yao He[5], Rohil Dhaliwal[1], Reilly Osadchey[9], Mrinal Shekhar[10], Ralph Mazitschek[5,7,8], Matthew G. Rees[5], Jennifer A. Roth[5], Qiang Cui[9,11,12], James D. Griffin[6], Brian B. Liau[4,5], and Amit Choudhary[1,2,3,*]

[1]Chemical Biology and Therapeutics Science Program, Broad Institute of MIT and Harvard, Cambridge, MA 02142, USA
[2]Divisions of Renal Medicine and Engineering, Brigham and Women's Hospital, Boston, MA 02115, USA
[3]Department of Medicine, Harvard Medical School, Boston, MA 02115, USA
[4]Department of Chemistry and Chemical Biology, Harvard University, Cambridge, MA 02138, USA
[5]Broad Institute of MIT and Harvard, Cambridge, MA 02142, USA
[6]Department of Medical Oncology, Dana-Farber Cancer Institute, Boston, MA 02215, USA
[7]Center for Systems Biology, Massachusetts General Hospital, Boston, MA 02114, USA
[8]Harvard T.H. Chan School of Public Health, Boston, MA 02115, USA
[9]Department of Chemistry, Boston University, Boston, MA 02215, USA
[10]Center for the Development of Therapeutics, Broad Institute of MIT and Harvard, Cambridge, MA 02142, USA
[11]Department of Physics, Boston University, Boston, MA 02215, USA
[12]Department of Biomedical Engineering, Boston University, Boston, MA 02215, USA

¶These authors contributed equally to this work and are listed arbitrarily. They will put their name first in their curriculum vitae citations or elsewhere.

*To whom correspondence should be addressed:

Amit Choudhary
Chemical Biology and Therapeutics Science Program
Broad Institute of MIT and Harvard
415 Main Street, Rm 3012
Cambridge, MA 02142
Phone: (617)714-7445
Fax: (617)715-8969
Email: achoudhary@bwh.harvard.edu

Table of contents

1. Compound characterization

Synthesis details and characterization of compounds **3**

^{1}H and ^{13}C NMR spectra **19**

2. Supporting References **37**

1. COMPOUND CHARACTERIZATION

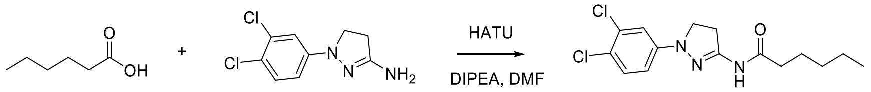

Compound 1 (VS772): N-(1-(3,4-dichlorophenyl)-4,5-dihydro-1H-pyrazol-3-yl)hexanamide. The synthesis and characterization of VS772 were previously reported (ref[1]). In brief, hexanoic acid (23 mg, 200 µmol), O-(7-Azabenzotriazol-1-yl)-N,N,N',N'-tetramethyl-uronium hexafluorophosphate (HATU) (100 mg, 260 µmol), and DIPEA (100 uL, 570 µmol) were combined in DMF (2 mL) and stirred at room temperature for 30 min. 1-(3,4-Dichlorophenyl)-4,5-dihydro-1H-pyrazol-3-amine 28 (46 mg) was added, and the reaction mixture was stirred at room temperature overnight. The solvent was removed under reduced pressure, and the residue was purified via flash column chromatography (Hex:EtOAc gradient from 100:0 to 70:30).

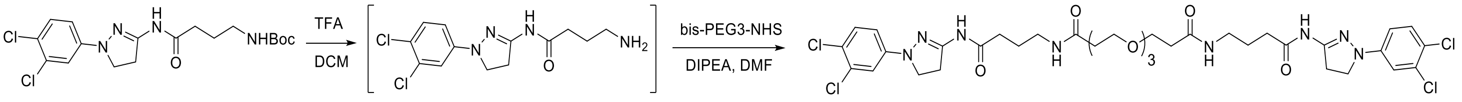

Compound 2 (VS1143): 4,4'-((3,3'-((oxybis(ethane-2,1-diyl))bis(oxy))bis(propanoyl))bis(azanediyl))bis(N-(1-(3,4-dichlorophenyl)-4,5-dihydro-1H-pyrazol-3-yl)butanamide). Tert-butyl (4-((1-(3,4-dichlorophenyl)-4,5-dihydro-1H-pyrazol-3-yl)amino)-4-oxobutyl)carbamate (ref[1]) (21 mg, 51 µmol) was dissolved in DCM (2 mL) and treated with TFA (500 µL). The reaction mixture was stirred at room temperature for 30 min and concentrated under reduced pressure. To the oil residue, bis-PEG3-NHS ester (11.1 mg, 25 µmol) in DMF (1 mL) was added, followed by DIPEA (50 µL). The reaction mixture was stirred at room temperature for 30 min, concentrated under reduced pressure, and purified by HPLC, affording the desired product VS1143 as a white powder (12 mg, 57% yield). ^{1}H NMR (400 MHz, $CDCl_3$ + CD_3OD (1:1)) δ 7.20 (d, J = 8.9 Hz, 2H), 6.99 (d, J = 2.7 Hz, 2H), 6.70 (dd, J = 9.1, 2.5 Hz, 2H), 3.76 – 3.65 (m, 8H), 3.60 (s, 9H), 3.40 (t, J = 9.8 Hz, 4H), 3.22 (t, J = 6.8 Hz, 4H), 2.42 (t, J = 6.1 Hz, 4H), 2.33 (t, J = 7.5 Hz, 4H), 1.80 ppm (p, J = 7.1 Hz, 4H). ^{13}C NMR (400 MHz, $CDCl_3$ + CD_3OD (1:1)) δ 172.34, 172.01, 148.96, 146.29, 132.02, 129.91, 120.24, 113.61, 111.70, 69.85, 69.72, 66.74, 38.11, 36.21, 33.12, 32.29, 24.64 ppm. HRMS (ESI-TOF): calculated for $C_{36}H_{47}Cl_4N_8O_7$ (M+H): 842.2244, found: 842.2243.

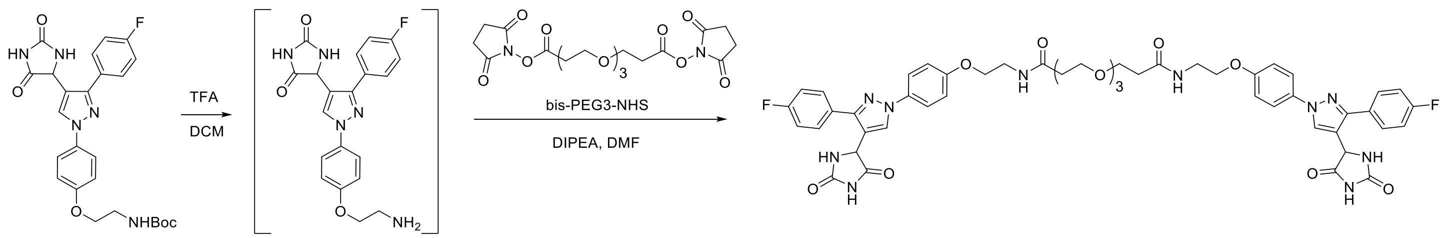

Compound 3 (VS1524): 3,3'-((oxybis(ethane-2,1-diyl))bis(oxy))bis(N-(2-(4-(4-(2,5-dioxoimidazolidin-4-yl)-3-(4-fluorophenyl)-1H-pyrazol-1-yl)phenoxy)ethyl)propanamide). *Tert*-butyl (2-(4-(4-(2,5-dioxoimidazolidin-4-yl)-3-(4-fluorophenyl)-1H-pyrazol-1-yl)phenoxy)ethyl)carbamate (ref[1]) (25 mg, 51 µmol) was dissolved in DCM (2 mL) and treated with TFA (500 µL). The reaction mixture was stirred at room temperature for 30 min and concentrated under reduced pressure. To the oil residue, bis-PEG3-NHS ester (11.1 mg, 25 µmol) in DMF (1 mL) was added, followed by DIPEA (50 µL). The reaction mixture was stirred at room temperature for 30 min, concentrated under reduced pressure, and purified by HPLC, affording the desired product VS1524 as a white powder (15 mg, 60% yield). ^{1}H NMR (400 MHz, $CDCl_3$ + CD_3OD (1:1)) δ 8.03 (s, 2H), 7.81 – 7.67 (m, 4H), 7.66 – 7.50 (m, 5H), 7.13 (t, J = 8.7 Hz, 4H), 7.03 – 6.92 (m, 4H), 5.21 (d, J = 1.4 Hz, 2H), 4.03 (t, J = 5.4 Hz, 4H), 3.69 (t, J = 6.0 Hz, 4H), 3.62 – 3.47 (m, 12H), 2.45 ppm (t, J = 6.0 Hz, 4H). ^{13}C NMR (101 MHz, $CDCl_3$ + CD_3OD (1:1)) δ 173.45, 165.00, 162.54, 158.74, 158.48, 152.06, 134.06, 131.15, 131.07, 128.89, 128.35, 121.70, 116.17, 116.11, 115.96, 115.79, 70.80, 70.70, 67.67, 67.39, 54.91, 39.47, 37.06 ppm. HRMS (ESI-TOF): calculated for $C_{50}H_{50}F_2N_{10}O_{11}Na$ (M+Na): 1027.3521, found: 1027.3522.

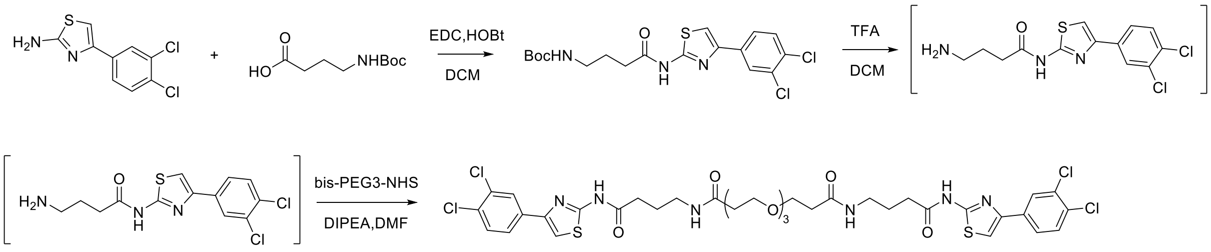

Compound 4 (VS1189): 4,4'-((3,3'-((oxybis(ethane-2,1-diyl))bis(oxy))bis(propanoyl))bis(azanediyl))bis(N-(4-(3,4-dichlorophenyl)thiazol-2-yl)butanamide). 4-(3,4-dichlorophenyl)thiazol-2-amine (24 mg, 0.1 mmol) was combined with N-Boc gamma-amino butyric acid (22 mg, 0.11 mmol), 1-Ethyl-3-(3-dimethylaminopropyl)carbodiimide (EDC) (77 mg, 0.4 mmol), and 1-Hydroxybenzotriazole (61 mg, 0.45 mmol) in 3 ml dichloromethane. Reaction was stirred at room temperature overnight (16 h), and the product isolated following previously reported procedures (ref[2]). Tert-butyl (4-((4-(3,4-dichlorophenyl)thiazol-2-yl)amino)-4-oxobutyl)carbamate (43 mg, 100 μmol) was dissolved in DCM (4 mL) and 1 mL TFA was added. The reaction mixture was stirred at room temperature for 30 min and concentrated under reduced pressure. To the oil residue, bis-PEG3-NHS ester (22.2 mg, 50 μmol) in DMF (2 mL) was added, followed by DIPEA (100 μL). The reaction mixture was stirred at room temperature for 30 min, concentrated under reduced pressure, and purified by HPLC, affording the desired product VS1189 as an off-white powder (7.9 mg, 18% yield). ^{1}H NMR (400 MHz, DMSO-*d*$_6$) δ 12.21 (s, 2H), 8.11 (d, *J* = 2.0 Hz, 2H), 7.91 – 7.82 (m, 4H), 7.79 (s, 2H), 7.67 (d, *J* = 8.4 Hz, 2H), 3.58 (t, *J* = 6.5 Hz, 4H), 3.48 – 3.42 (m, 8H), 3.08 (q, *J* = 6.6 Hz, 4H), 2.45 (t, *J* = 7.4 Hz, 4H), 2.29 (t, *J* = 6.5 Hz, 4H), 1.73 (p, *J* = 7.2 Hz, 4H). ^{13}C NMR (101 MHz, DMSO-*d*$_6$) 171.36, 170.04, 158.21, 146.11, 134.87, 131.53, 130.98, 129.96, 127.30, 125.65, 109.95, 69.64, 69.51, 66.83, 37.86, 36.18, 32.34, 24.67. HRMS (ESI-TOF): calculated for $C_{36}H_{40}Cl_4N_6O_7S_2$ (M+H): 873.1227, found: 873.1220.

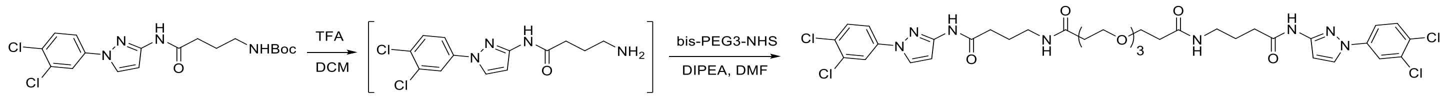

Compound 5 (VS1144): 4,4'-((3,3'-((oxybis(ethane-2,1-diyl))bis(oxy))bis(propanoyl))bis(azanediyl))bis(N-(1-(3,4-dichlorophenyl)-1H-pyrazol-3-yl)butanamide). Tert-butyl (4-((1-(3,4-dichlorophenyl)-1H-pyrazol-3-yl)amino)-4-oxobutyl)carbamate (ref[1]) (41 mg, 100 μmol) was dissolved in DCM (4 mL) and treated with TFA (1 mL). The reaction mixture was stirred at room temperature for 30 min and concentrated under reduced pressure. To the oil residue, bis-PEG3-NHS ester (22.2 mg, 50 μmol) in DMF (2 mL) was added, followed by DIPEA (100 μL). The reaction mixture was stirred at room temperature for 30 min, concentrated under reduced pressure, and purified by HPLC, affording the desired product VS1144 as a white powder (20 mg, 48% yield). ^{1}H NMR (400 MHz, CD_3OD) δ 8.07 (d, *J* = 2.6 Hz, 2H), 7.86 (d, *J* = 2.5 Hz, 2H), 7.57 (dd, *J* = 8.8, 2.6 Hz, 2H), 7.47 (d, *J* = 8.8 Hz, 2H), 6.77 (d, *J* = 2.6 Hz, 2H), 3.63 (t, *J* = 6.1 Hz, 4H), 3.52 (tt, *J* = 5.4, 2.7 Hz, 9H), 3.19 (t, *J* = 6.8 Hz, 5H), 2.35 (t, *J* = 6.2 Hz, 9H), 1.80 ppm (p, *J* = 7.1 Hz, 4H). ^{13}C NMR (400 MHz, CD_3OD) δ174.15, 173.47, 150.69, 140.68, 134.17, 132.24, 129.97, 129.09, 120.81, 118.39, 102.00, 71.44, 71.34, 68.26, 39.75, 37.73, 34.53, 26.41. ppm. HRMS (ESI-TOF): calculated for $C_{36}H_{43}Cl_4N_8O_7$ (M+H): 841.1974, found 841.1971.

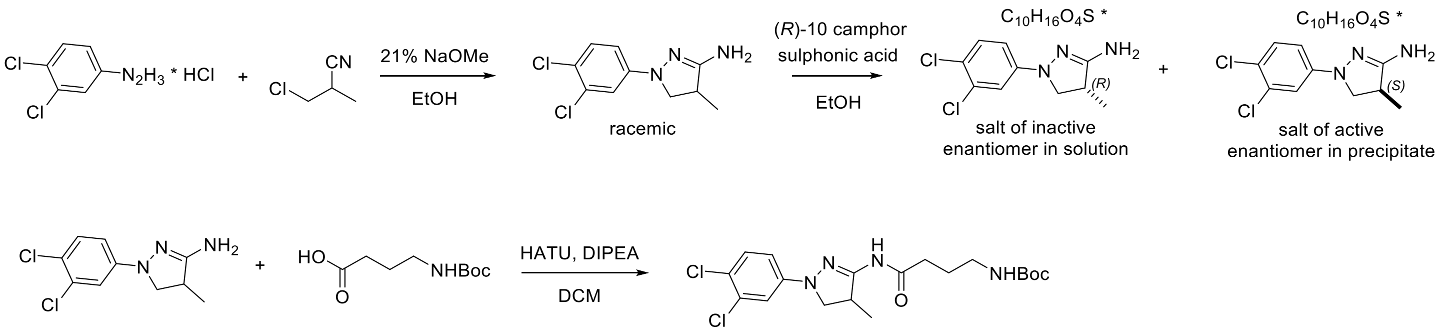

VS1060: 1-(3,4-dichlorophenyl)-4-methyl-4,5-dihydro-1H-pyrazol-3-amine, separation of enantiomers and linker attachment. To a suspension of 3,4-dichlorophenylhydrazine hydrochloride (2.1 g, 10 mmol) in EtOH (8 mL) at room temperature, sodium methoxide in MeOH (21 w/w %, 6.4 mL, 24 mmol) was added dropwise. To the mixture, 3-chloro-2-methylpropionitrile (1.1 g, 11 mmol) in 2.5 mL EtOH was added dropwise and the mixture was stirred at 85 °C overnight. The next day, the resulting suspension was evaporated to remove EtOH, water was added, and the yellowish solid was filtered, washed, and dried to provide the desired product (1.75 g, 90% yield). Characterization data of the resulting product matches the data reported for 1-(3,4-dichlorophenyl)-4-methyl-4,5-dihydro-1H-pyrazol-3-amine (ref[3]). The enantiomers of 1-(3,4-dichlorophenyl)-4-methyl-4,5-dihydro-1H-pyrazol-3-amine were separated by recrystallization of diastereomers formed in the reaction with (*R*)-10 camphor sulphonic acid. More specifically, 1-(3,4-dichlorophenyl)-4-methyl-4,5-dihydro-1H-pyrazol-3-amine (1.2 g, 5 mmol) was dissolved in EtOH (12 mL) and treated with (*R*)-10 camphor sulphonic acid (1.2 g, 5 mmol). The mixture was heated at 50 °C for 30 min and cooled down to room temperature. After 1h the precipitate was collected via filtration (solid residue contains active (*S*)-enantiomer; absolute stereochemistry assigned based on reported activity data [ref[3]]) and separated from the filtrate (solution contains inactive (*R)*-enantiomers) that is later concentrated under reduced pressure. Salts of separated enantiomers were dissolved in water, neutralized by NaOH, extracted by DCM, washed with brine, and concentrated under reduced pressure affording active and inactive enantiomers of 1-(3,4-dichlorophenyl)-4-methyl-4,5-dihydro-1H-pyrazol-3-amine used in the following steps without further characterization. Coupling of racemic and active/inactive enantiomers 1-(3,4-dichlorophenyl)-4-methyl-4,5-dihydro-1H-pyrazol-3-amine with N-Boc gamma-amino butyric acid was performed using the earlier reported method affording racemic and active/inactive enantiomers of tert-butyl (4-((1-(3,4-dichlorophenyl)-4-methyl-4,5-dihydro-1H-pyrazol-3-yl)amino)-4-oxobutyl)carbamate used in the next steps (ref[2]).

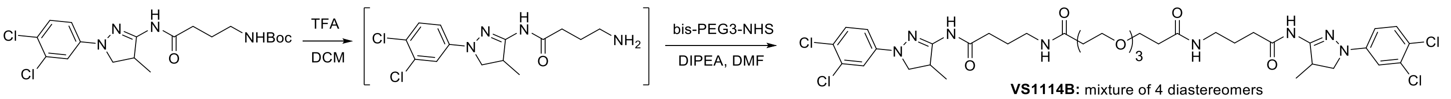

Compound 6 (VS1114B): 4,4'-((3,3'-((oxybis(ethane-2,1-diyl))bis(oxy))bis(propanoyl))bis(azanediyl)) bis(N-(1-(3,4-dichlorophenyl)-4-methyl-4,5-dihydro-1H-pyrazol-3-yl)butanamide). Racemic tert-butyl (4-((1-(3,4-dichlorophenyl)-4-methyl-4,5-dihydro-1H-pyrazol-3-yl)amino)-4-oxobutyl)carbamate (42.9 mg, 100 µmol) was dissolved in DCM (4 mL) and treated with TFA (1 mL). The reaction mixture was stirred at room temperature for 30 min and concentrated under reduced pressure. To the oil residue, bis-PEG3-NHS ester (22.2 mg, 50 µmol) in DMF (2 mL) was added, followed by DIPEA (100 µL). The reaction mixture was stirred at room temperature for 30 min, concentrated under reduced pressure, and purified by HPLC, affording the desired product VS1144 as a white powder (25 mg, 57% yield). ^{1}H NMR (400 MHz, $CDCl_3$ + CD_3OD (1:1)) δ 7.78 (t, J = 5.8 Hz, 2H), 7.22 (d, J = 8.8 Hz, 2H), 7.02 (d, J = 2.6 Hz, 2H), 6.75 (s, 2H), 3.87 (s, 2H), 3.77 – 3.66 (m, 6H), 3.61 (s, 8H), 3.53 (dd, J = 9.5, 4.3 Hz, 2H), 3.24 (q, J = 6.5 Hz, 4H), 2.44 (t, J = 6.1 Hz, 4H), 2.37 (t, J = 7.5 Hz, 4H), 1.83 (p, J = 7.1 Hz, 4H), 1.25 (d, J = 6.9 Hz, 6H). 13C NMR (101 MHz, $CDCl_3$ + CD_3OD (1:1)) δ 173.43, 172.75, 153.61, 147.29, 133.06, 130.97, 121.42, 114.78, 112.87, 70.90, 70.77, 67.80, 56.75, 39.97, 39.19, 37.25, 34.19, 25.80, 17.71. HRMS (ESI-TOF): calculated for $C_{38}H_{51}Cl_4N_8O_7$ (M+H): 873.2600, found: 873.2598.

Compound 7 (VS1155): (*R*,*R*)-4,4'-((3,3'-((oxybis(ethane-2,1-diyl))bis(oxy))bis(propanoyl))bis(azanediyl)) bis(N-(1-(3,4-dichlorophenyl)-4-methyl-4,5-dihydro-1H-pyrazol-3-yl)butanamide). Using **the** inactive enantiomer of tert-butyl (4-((1-(3,4-dichlorophenyl)-4-methyl-4,5-dihydro-1H-pyrazol-3-yl)amino)-4-oxobutyl)carbamate and following the procedure for VS1114B, the desired product VS1155 was obtained (27 mg, 62%). ^{1}H and ^{13}C NMR data matched that reported for VS1114B. HRMS (ESI-TOF): calculated for $C_{38}H_{51}Cl_4N_8O_7$ (M+H): 873.2600, found: 873.2604.

Compound 8 (VS1156): (*S*,*S*)-4,4'-((3,3'-((oxybis(ethane-2,1-diyl))bis(oxy))bis(propanoyl))bis(azanediyl)) bis(N-(1-(3,4-dichlorophenyl)-4-methyl-4,5-dihydro-1H-pyrazol-3-yl)butanamide). Using the active enantiomer of tert-butyl (4-((1-(3,4-dichlorophenyl)-4-methyl-4,5-dihydro-1H-pyrazol-3-yl)amino)-4-oxobutyl)carbamate and following the procedure for VS1114B, the desired product VS1156 was obtained (25 mg, 57%). ^{1}H and ^{13}C NMR data matched that reported for VS1114B. HRMS (ESI-TOF): calculated for $C_{38}H_{51}Cl_4N_8O_7$ (M+H): 873.2600, found: 873.2604.

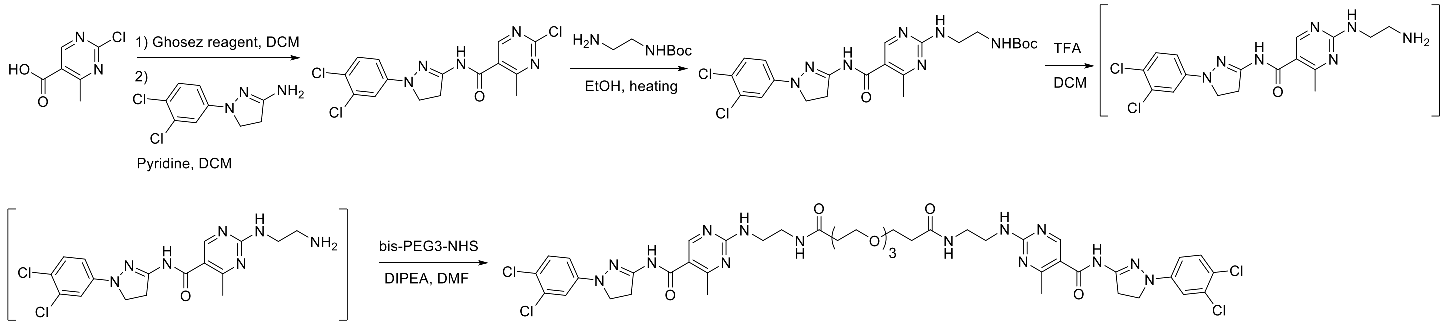

VS1065: 2-chloro-N-(1-(3,4-dichlorophenyl)-4,5-dihydro-1H-pyrazol-3-yl)-4-methylpyrimidine-5-carboxamide. 2-chloro-4-methylpyrimidine-5-carboxylic acid (120 mg, 0.7 mmol) was dissolved in DCM (6 mL) and treated with Ghosez reagent (180 µL, 1.4 mmol). The reaction mixture was stirred at room temperature for 1 hour, and 1-(3,4-dichlorophenyl)-4,5-dihydro-1H-pyrazol-3-amine (138 mg, 0.6 mmol) was added, followed by pyridine (180 µL). The reaction mixture was stirred at room temperature overnight, quenched with an aqueous $NaHCO_3$ solution, extracted with DCM, and concentrated under reduced pressure. The residue was purified by flash column chromatography (DCM: EtOAc gradient from 100:0 to 70:30), affording the desired product as a yellowish solid (45 mg, 21% yield). ^1H NMR (400 MHz, $CDCl_3$ + CD_3OD (1:1)) δ 8.67 (s, 1H), 7.24 (d, J = 8.8 Hz, 1H), 7.05 (d, J = 2.7 Hz, 1H), 6.77 (dd, J = 8.9, 2.7 Hz, 1H), 3.81 (t, J = 10.0 Hz, 2H), 3.56 (t, J = 10.0 Hz, 2H), 2.67 ppm (s, 3H). ^{13}C NMR (101 MHz, $CDCl_3$ + CD_3OD (1:1)) δ 170.66, 164.35, 161.94, 158.35, 149.44, 147.01, 133.16, 131.02, 128.25, 121.72, 114.79, 112.86, 49.31, 33.30, 22.89 ppm. HRMS (ESI-TOF): calculated for $C_{15}H_{13}Cl_3N_5O$ (M+H): 386.0151, found: 386.0148.

VS1067: tert-butyl (2-((5-((1-(3,4-dichlorophenyl)-4,5-dihydro-1H-pyrazol-3-yl)carbamoyl)-4-methylpyrimidin-2-yl)amino)ethyl)carbamate. 2-chloro-N-(1-(3,4-dichlorophenyl)-4,5-dihydro-1H-pyrazol-3-yl)-4-methylpyrimidine-5-carboxamide (VS1065, 22 mg, 60 µmol) and tert-butyl (2-aminoethyl)carbamate were dissolved in ethanol (0.5 mL) and heated at 70 ºC overnight. The next day, the solvent was removed under reduced pressure, and the residue was purified via flash column chromatography (DCM: MeOH gradient from 100:0 up to 95:5), affording the desired product as a yellowish solid (22 mg, 73% yield). ^1H NMR (400 MHz, CD_3OD) δ 8.43 (s, 1H), 7.27 (d, J = 8.9 Hz, 1H), 7.12 (d, J = 2.7 Hz, 1H), 6.83 (dd, J = 8.9, 2.7 Hz, 1H), 3.78 (t, J = 10.1 Hz, 2H), 3.62 – 3.43 (m, 4H), 3.27 (t, J = 6.1 Hz, 2H), 2.51 (s, 3H), 1.43 (s, 9H). ^{13}C NMR (101 MHz, CD_3OD) δ 167.20, 163.47, 158.89, 158.65, 151.19, 148.15, 133.43, 131.44, 121.41, 118.46, 115.07, 113.36, 80.14, 49.49, 42.29, 40.99, 33.77, 28.75, 23.55. HRMS (ESI-TOF): calculated for $C_{22}H_{28}Cl_2N_7O_3$ (M+H): 508.1625, found: 508.1628.

Compound 9 (VS1142): 2,2'-((4,16-dioxo-7,10,13-trioxa-3,17-diazanonadecane-1,19-diyl)bis(azanediyl)) bis(N-(1-(3,4-dichlorophenyl)-4,5-dihydro-1H-pyrazol-3-yl)-4-methylpyrimidine-5-carboxamide). Tert-butyl (2-((5-((1-(3,4-dichlorophenyl)-4,5-dihydro-1H-pyrazol-3-yl)carbamoyl)-4-methylpyrimidin-2-

yl)amino)ethyl)carbamate (VS1067) (22 mg, 43 µmol) was dissolved in DCM (1 mL) and treated with TFA (0.25 mL). The reaction mixture was stirred at room temperature for 30 min and concentrated under reduced pressure. To the oil residue, bis-PEG3-NHS ester (9.5 mg, 22 µmol) in DMF (1 mL) was added, followed by DIPEA (50 µL). The reaction mixture was stirred at room temperature for 30 min, concentrated under reduced pressure, and purified by HPLC, affording the desired product VS1142 as a white powder (10 mg, 23% yield). ^{1}H NMR (400 MHz, $CDCl_3$ + CD_3OD (1:1)) δ 8.41 (d, J = 4.5 Hz, 3H), 7.59 (s, 3H), 7.24 (d, J = 8.8 Hz, 2H), 7.06 (d, J = 2.7 Hz, 2H), 6.77 (dd, J = 8.8, 2.7 Hz, 2H), 3.78 (t, J = 9.9 Hz, 4H), 3.71 (t, J = 6.1 Hz, 4H), 3.61 (hept, J = 2.8 Hz, 8H), 3.58 – 3.50 (m, 8H), 3.44 (dd, J = 6.7, 5.1 Hz, 4H), 2.52 (s, 6H), 2.44 ppm (t, J = 6.1 Hz, 4H). ^{13}C NMR (400 MHz, $CDCl_3$ + CD_3OD (1:1)) δ 173.64, 166.41, 162.67, 158.25, 150.38, 147.26, 133.11, 130.98, 121.44, 114.75, 112.81, 70.85, 70.76, 67.72, 41.41, 39.65, 37.26, 33.39 ppm. HRMS (ESI-TOF): calculated for $C_{44}H_{53}Cl_4N_{14}O_7$ (M+H): 1031.2941, found: 1031.2946.

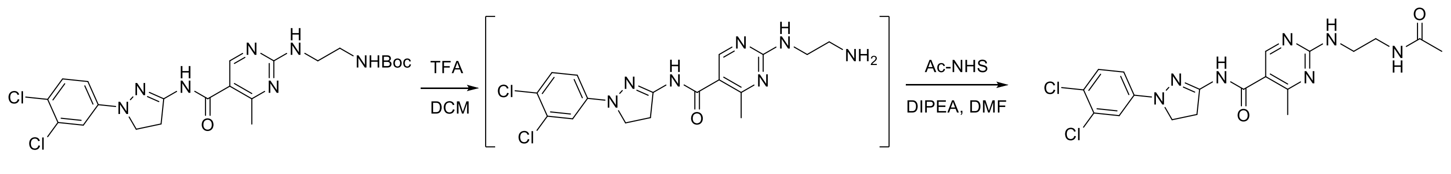

BRD6501: 2-((2-acetamidoethyl)amino)-N-(1-(3,4-dichlorophenyl)-4,5-dihydro-1H-pyrazol-3-yl)-4-methylpyrimidine-5-carboxamide. Tert-butyl (2-((5-((1-(3,4-dichlorophenyl)-4,5-dihydro-1H-pyrazol-3-yl)carbamoyl)-4-methylpyrimidin-2-yl)amino) ethyl)carbamate (VS1067) (22 mg, 43 µmol) was dissolved in DCM (1 mL) and treated with TFA (0.25 mL). The reaction mixture was stirred at room temperature for 30 min and concentrated under reduced pressure. To the oil residue, acetic acid N-hydroxysuccinimide ester (7.9 mg, 50 µmol) in DMF (1 mL) was added, followed by DIPEA (50 µL). The reaction mixture was stirred at room temperature for 30 min, concentrated under reduced pressure, and purified by HPLC, affording the desired product VS1148 as a white powder (12 mg, 62% yield). ^{1}H NMR (400 MHz, $CDCl_3$ + CD_3OD (1:1)) δ 8.41 (s, 1H), 7.61 (s, 1H), 7.23 (d, J = 8.8 Hz, 1H), 7.06 (d, J = 2.6 Hz, 1H), 6.77 (dd, J = 8.9, 2.6 Hz, 1H), 3.77 (t, J = 9.9 Hz, 2H), 3.60 – 3.46 (m, 4H), 3.39 (t, J = 6.0 Hz, 2H), 2.51 (s, 3H), 1.93 ppm (s, 3H). ^{13}C NMR (400 MHz, $CDCl_3$ + CD_3OD (1:1)) δ 173.16, 166.51, 162.67, 158.28, 150.42, 147.32, 133.12, 131.00, 121.41, 114.76, 112.86, 41.38, 39.80, 33.42, 22.67. HRMS (ESI-TOF): calculated for $C_{19}H_{22}Cl_2N_7O_2$ (M+H): 450.1207, found: 450.1207.

Synthesis of compounds with different linker lengths (PEG0–3):

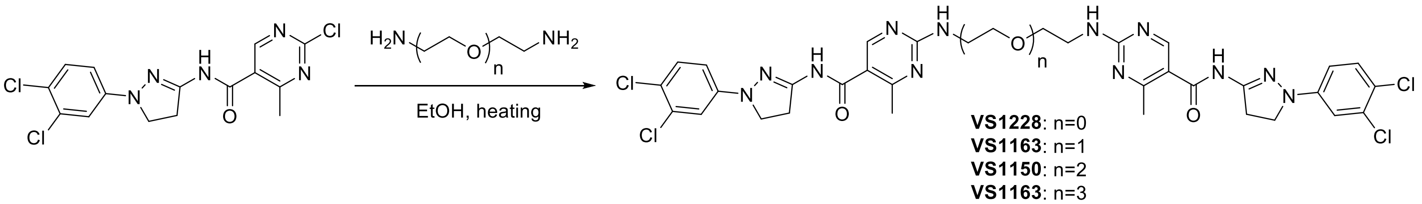

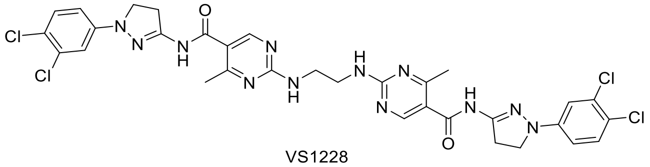

VS1228

2,2'-(ethane-1,2-diylbis(azanediyl))bis(N-(1-(3,4-dichlorophenyl)-4,5-dihydro-1H-pyrazol-3-yl)-4-methylpyrimidine-5-carboxamide) (VS1228). 2-chloro-N-(1-(3,4-dichlorophenyl)-4,5-dihydro-1H-pyrazol-3-yl)-4-methylpyrimidine-5-carboxamide (VS1065, 20 mg, 52 μmol) and ethylenediamine (2 mg, 33 μmol) were dissolved in 1 mL EtOH, and DIPEA was added (20 μL). The reaction was heated at 70 ºC overnight. The solvent was removed under reduced pressure, and the oil residue was purified via HPLC, affording the desired product VS1228 as a white solid (6.6 mg, 35% yield). ^{1}H NMR (400 MHz, DMSO-*d*$_6$) δ 10.97 (s, 2H), 8.56 – 8.38 (m, 2H), 7.80 – 7.61 (m, 2H), 7.41 (d, *J* = 8.8 Hz, 2H), 7.08 (d, *J* = 2.6 Hz, 2H), 6.85 (dd, *J* = 9.0, 2.7 Hz, 2H), 3.75 (t, *J* = 9.8 Hz, 4H), 3.60 – 3.39 (m, 8H), 2.42 (s, 6H).^{13}C NMR (101 MHz, DMSO-*d*$_6$) δ 167.54, 164.89, 161.78, 158.09, 150.97, 146.55, 131.33, 130.66, 118.41, 116.22, 113.21, 112.35, 47.92, 32.75, 23.56, 23.06. HRMS (ESI-TOF): calculated for $C_{32}H_{30}Cl_4N_{12}O_2$ (M+H): 755.1442, found: 757.1404.

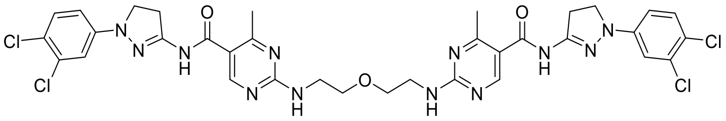

VS1163

2,2'-((oxybis(ethane-2,1-diyl))bis(azanediyl))bis(N-(1-(3,4-dichlorophenyl)-4,5-dihydro-1H-pyrazol-3-yl)-4-methylpyrimidine-5-carboxamide) (VS1163). 2-chloro-N-(1-(3,4-dichlorophenyl)-4,5-dihydro-1H-pyrazol-3-yl)-4-methylpyrimidine-5-carboxamide (VS1065, 20 mg, 52 μmol) and 2,2'-oxybis(ethan-1-amine) (2.7 mg, 26 μmol) were dissolved in 1 mL EtOH, and DIPEA was added (20 μL). The reaction was heated at 70 ºC overnight. The solvent was removed under reduced pressure and oil residue was purified via HPLC, affording the desired product VS1163 as a yellowish solid (8 mg, 38% yield). ^{1}H NMR (400 MHz, DMSO-*d*$_6$) δ 10.95 (s, 2H), 8.47 (s, 2H), 7.70 (s, 2H), 7.41 (d, J = 8.9 Hz, 2H), 7.08 (d, J = 2.6 Hz, 2H), 6.84 (dd, J = 8.9, 2.7 Hz, 2H), 3.75 (t, J = 9.8 Hz, 4H), 3.63 – 3.42 (m, 12H), 2.43 (s, 6H). ^{13}C NMR (101 MHz, DMSO-*d*$_6$) δ 165.38, 162.24, 158.73, 151.43, 147.03, 131.82, 131.12, 118.90, 113.69, 112.82, 69.10, 55.38, 48.39, 33.23, 31.16 HRMS (ESI-TOF): calculated for $C_{34}H_{35}Cl_4N_{12}O_3$ (M+H): 801.1674, found: 801.1676.

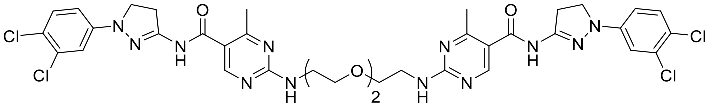

VS1150

BRD8833 (VS1150): 2,2'-(((ethane-1,2-diylbis(oxy))bis(ethane-2,1-diyl))bis(azanediyl))bis(N-(1-(3,4-dichlorophenyl)-4,5-dihydro-1H-pyrazol-3-yl)-4-methylpyrimidine-5-carboxamide). 2-chloro-N-(1-(3,4-dichlorophenyl)-4,5-dihydro-1H-pyrazol-3-yl)-4-methylpyrimidine-5-carboxamide (VS1065, 40 mg, 104 µmol) and 2,2'-(ethane-1,2-diylbis(oxy))bis(ethan-1-amine) (7.5 mg, 51 µmol) were dissolved in 1 mL EtOH, and DIPEA was added (20 µL). The reaction was heated at 70 ºC overnight. The solvent was removed under reduced pressure, and the oil residue was purified via HPLC, affording the desired product VS1150 as a yellowish solid (20 mg, 44% yield). ^{1}H NMR (400 MHz, DMSO-*d$_6$*) δ 8.46 (d, J = 15.5 Hz, 2H), 7.63 (d, J = 18.0 Hz, 2H), 7.41 (d, J = 8.8 Hz, 2H), 7.08 (d, J = 2.7 Hz, 2H), 6.84 (dd, J = 8.9, 2.7 Hz, 2H), 3.75 (t, J = 9.8 Hz, 4H), 3.63 – 3.39 (m, 16H), 2.51 (p, J = 1.8 Hz, 6H), 2.43 ppm (d, J = 11.0 Hz, 4H). ^{13}C NMR (101 MHz, DMSO-*d$_6$*) δ 167.61, 164.91, 161.74, 158.14, 151.01, 146.55, 131.36, 130.68, 118.42, 116.43, 113.22, 112.36, 69.61, 68.77, 47.93, 40.40, 32.78, 30.74 ppm. HRMS (ESI-TOF): calculated for $C_{36}H_{39}Cl_4N_{12}O_4$ (M+H): 845.1936, found: 845.1937.

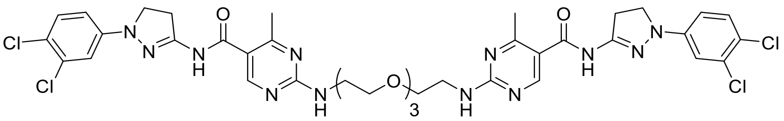

VS1186

2,2'-((((oxybis(ethane-2,1-diyl))bis(oxy))bis(ethane-2,1-diyl))bis(azanediyl))bis(N-(1-(3,4-dichlorophenyl)-4,5-dihydro-1H-pyrazol-3-yl)-4-methylpyrimidine-5-carboxamide) (VS1186). 2-chloro-N-(1-(3,4-dichlorophenyl)-4,5-dihydro-1H-pyrazol-3-yl)-4-methylpyrimidine-5-carboxamide (VS1065, 15 mg, 39 μmol) and 2,2'-((oxybis(ethane-2,1-diyl))bis(oxy))bis(ethan-1-amine) (3.8 mg, 20 μmol) were dissolved in 1 mL EtOH, and DIPEA was added (20 μL). The reaction was heated at 70 ºC overnight. The solvent was removed under reduced pressure and the oil residue was purified via HPLC, affording the desired product VS1186 as a yellowish solid (12 mg, 68% yield). ^{1}H NMR (400 MHz, DMSO-d_6) δ 8.47 (s, 2H), 7.59 (s, 2H), 7.40 (d, *J* = 8.9 Hz, 2H), 7.17 – 7.00 (m, 2H), 6.84 (d, *J* = 9.0 Hz, 2H), 3.75 (t, *J* = 9.9 Hz, 4H), 3.51 (m, 20H), 2.43 (s, 4H), 2.08 (s, 6H). ^{13}C NMR (101 MHz, DMSO-d6) ^{13}C NMR (101 MHz, DMSO-d_6) δ 167.54, 164.88, 161.72, 158.15, 150.93, 146.53, 131.32, 130.62, 118.40, 118.04, 113.20, 112.32, 69.76, 69.59, 68.72, 54.89, 47.89, 40.38, 40.15, 39.94, 39.73, 39.52, 39.31, 39.10, 38.89, 32.73. HRMS (ESI-TOF): calculated for $C_{38}H_{43}Cl_4N_{12}O_5$ (M+H): 889.2198, found: 889.2198.

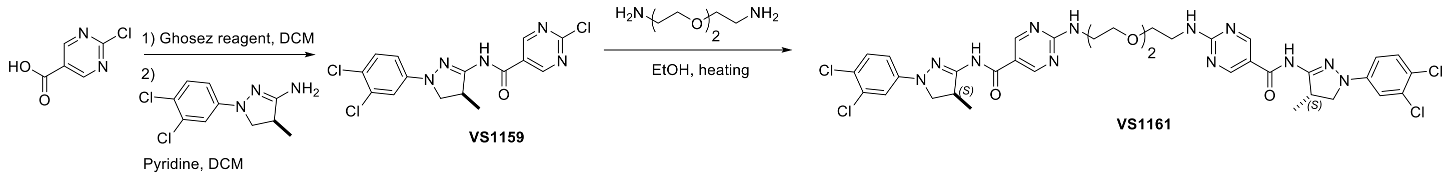

(*S*)-2-chloro-N-(1-(3,4-dichlorophenyl)-4-methyl-4,5-dihydro-1H-pyrazol-3-yl)pyrimidine-5-carboxamide (VS1159). 2-chloropyrimidine-5-carboxylic acid (180 mg, 1.1 mmol) was dissolved in DCM (10 mL) and treated with Ghosez reagent (180 µL, 2.1 mmol). The reaction mixture was stirred at room temperature for 1 hour and (*S*)-1-(3,4-dichlorophenyl)-4-methyl-4,5-dihydro-1H-pyrazol-3-amine (VS1060) (225 mg, 0.9 mmol) was added, followed by pyridine (270 µL). The reaction mixture was stirred at room temperature overnight, quenched with an aqueous $NaHCO_3$ solution, extracted with DCM, and concentrated under reduced pressure. The residue was purified by flash column chromatography (DCM: EtOAc gradient from 100:0 to 70:30) affording the desired product as a yellowish solid (14 mg, 4.1% yield). ^{1}H NMR (400 MHz, $CDCl_3$ + CD_3OD (1:1)) δ 9.14 (s, 2H), 7.27 (d, J = 8.8 Hz, 1H), 7.09 (d, J = 2.6 Hz, 1H), 6.81 (dd, J = 8.8, 2.7 Hz, 1H), 4.08 (dtd, J = 14.2, 7.0, 3.5 Hz, 1H), 3.83 (t, J = 10.0 Hz, 1H), 3.65 (dd, J = 9.5, 4.5 Hz, 1H), 1.34 ppm (d, J = 7.0 Hz, 3H). ^{13}C NMR (101 MHz, $CDCl_3$ + CD_3OD (1:1)) δ 164.01, 161.75, 159.89, 153.02, 146.72, 133.12, 130.97, 127.01, 121.82, 114.79, 112.82, 56.90, 39.91, 17.78. HRMS (ESI-TOF): calculated for $C_{15}H_{13}Cl_3N_5O$ (M+H): 386.0151, found: 386.0145.

BRD8834 (VS1161): 2,2'-(((ethane-1,2-diylbis(oxy))bis(ethane-2,1-diyl))bis(azanediyl))bis(N-((S)-1-(3,4-dichlorophenyl)-4-methyl-4,5-dihydro-1H-pyrazol-3-yl)pyrimidine-5-carboxamide). (*S*)-2-chloro-N-(1-(3,4-dichlorophenyl)-4-methyl-4,5-dihydro-1H-pyrazol-3-yl)pyrimidine-5-carboxamide (VS1159, 12 mg, 31 µmol) and 2,2'-(ethane-1,2-diylbis(oxy))bis(ethan-1-amine) (2 mg, 14 µmol) were dissolved in 0.5 mL EtOH, and DIPEA was added (10 µL). The reaction was heated at 70 ºC overnight. The solvent was removed under reduced pressure, and the oil residue was purified via HPLC, affording the desired product VS1161 as a yellowish solid (7 mg, 59% yield). ^{1}H NMR (400 MHz, $CDCl_3$ + CD_3OD (1:1)) δ 8.81 (s, 4H), 7.52 (s, 5H), 7.25 (d, *J* = 8.8 Hz, 2H), 7.06 (d, *J* = 2.7 Hz, 2H), 6.78 (dd, *J* = 8.9, 2.7 Hz, 2H), 3.84 – 3.74 (m, 2H), 3.72 – 3.65 (m, 8H), 3.59 (dd, *J* = 9.5, 4.6 Hz, 2H), 3.33 (s, 8H), 1.31 (d, *J* = 7.0 Hz, 6H). ^{13}C NMR (101 MHz, DMSO-d_6) δ 163.17, 162.91, 158.94, 154.37, 146.75, 131.73, 131.00, 119.22, 115.74, 113.81, 112.86, 69.92, 68.94, 55.77, 40.92, 17.35. HRMS (ESI-TOF): calculated for $C_{36}H_{39}Cl_4N_{12}O_4$ (M+H): 845.1936, found: 845.1935.

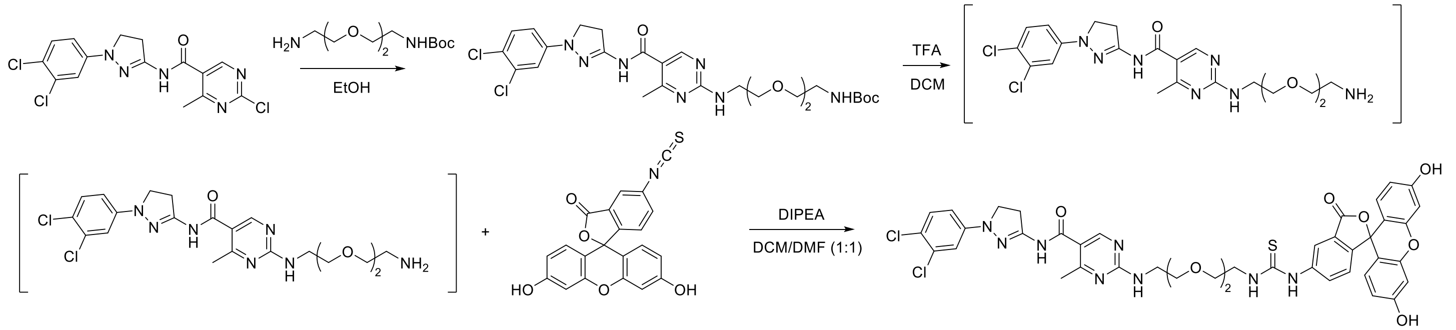

TR-FRET tracer (VS1231): N-(1-(3,4-dichlorophenyl)-4,5-dihydro-1H-pyrazol-3-yl)-2-((2-(2-(2-(3-(3',6'-dihydroxy-3-oxo-3H-spiro[isobenzofuran-1,9'-xanthen]-5-yl)thioureido)ethoxy)ethoxy)ethyl)amino) pyrimidine-5-carboxamide: Tert-butyl (2-(2-(2-aminoethoxy)ethoxy)ethyl)carbamate (30 mg, 0.12 mmol) is added to 2-chloro-N-(1-(3,4-dichlorophenyl)-4,5-dihydro-1H-pyrazol-3-yl)pyrimidine-5-carboxamide (VS1065) (45 mg, 0.12 mmol) in 1 mL EtOH. The reaction was heated at 70ºC overnight, and the solvent was removed under reduced pressure. The resulting oil was purified by flash chromatography (DCM:MeOH). The resulting tert-butyl (2-(2-((5-((1-(3,4-dichlorophenyl)-4,5-dihydro-1H-pyrazol-3-yl)carbamoyl)pyrimidin-2-yl)amino)ethoxy) -3-methoxypropyl)carbamate (40 mg, 0.07 mmol) was dissolved in 3 mL DCM, and 0.75 mL TFA was added. After 30 min, the reaction mixture was concentrated under reduced pressure, and the residue was dissolved in 1 mL of a 1:1 mixture of DCM and DMF. To this, 3',6'-dihydroxy-5-isothiocyanato-3H-spiro[isobenzofuran-1,9'-xanthen]-3-one (FITC-isothiocyanate) (30 mg, 0.077 mmol) and 100 µL of DIPEA were added. The reaction was stirred for 1 h at room temperature, the solvent removed under reduced pressure, and the residue purified via HPLC, affording the desired product VS1231 as an orange solid (8.3 mg, 8% yield). ^{1}H NMR (400 MHz, $CDCl_3$ + CD_3OD (1:1)) δ 8.43 (s, 1H), 8.12 (d, *J* = 2.0 Hz, 1H), 7.88 (d, *J* = 8.6 Hz, 1H), 7.25 (d, *J* = 8.8 Hz, 1H), 7.16 (d, *J* = 8.3 Hz, 1H), 7.07 (d, *J* = 2.7 Hz, 1H), 6.79 (dd, *J* = 8.9, 2.7 Hz, 1H), 6.72 – 6.63 (m, 4H), 6.54 (dd, *J* = 8.7, 2.4 Hz, 2H), 3.88 (s, 1H), 3.83 – 3.73 (m, 5H), 3.73 – 3.60 (m, 8H), 3.55 (t, *J* = 9.9 Hz, 2H), 2.53 (s, 3H). ^{13}C NMR (101 MHz, $CDCl_3$ + CD_3OD (1:1)) δ 181.43, 169.91, 168.90, 165.60, 161.69, 159.83, 157.53, 152.91, 149.59, 146.51, 140.90, 132.35, 130.94, 129.06, 127.66, 124.53, 120.64, 118.60, 117.24, 116.69, 113.97, 112.47, 112.10, 110.15, 102.57, 70.14, 70.08, 69.39, 44.23, 40.75, 39.68, 32.64, 29.48, 22.82. HRMS (ESI-TOF): calculated for $C_{42}H_{38}Cl_2N_8O_8S$ (M+H): 885.1982, found: 885.1969.

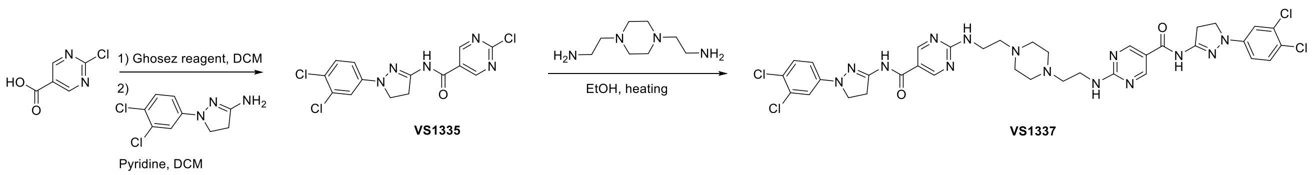

2-chloro-N-(1-(3,4-dichlorophenyl)-4,5-dihydro-1H-pyrazol-3-yl)pyrimidine-5-carboxamide (VS1335). 2-chloropyrimidine-5-carboxylic acid (400 mg, 2.5 mmol) was dissolved in DCM (10 mL) and treated with oxalyl chloride (0.4 mL) followed by 1 drop of DMF. The reaction mixture was stirred at room temperature for 1 hour, concentrated under reduced pressure, and redissolved in THF and DCM (1:1, 25 mL total), and treated with 1-(3,4-dichlorophenyl)-4,5-dihydro-1H-pyrazol-3-amine (460 mg, 2 mmol), followed by DIPEA (1 mL). The reaction mixture was stirred overnight, $NaHCO_3$ was added, and the product was extracted with DCM, washed with brine, concentrated under reduced pressure, and purified by flash column chromatography (DCM : EtOAc slow gradient from 100:0 to 90:10) affording the desired product as a yellowish solid (150 mg, 20% yield). ^{1}H NMR (400 MHz, Acetone-d6) δ 10.52 (s, 1H), 9.22 (d, *J* = 0.8 Hz, 2H), 7.30 (dd, *J* = 8.9, 0.8 Hz, 1H), 7.03 (d, *J* = 2.6 Hz, 1H), 6.79 (dt, *J* = 8.9, 1.6 Hz, 1H), 3.85 (t, *J* = 10.0 Hz, 2H), 3.58 (t, *J* = 10.0 Hz, 2H). ^{13}C NMR (101 MHz, Acetone-d6) δ 163.97, 161.99, 160.34, 150.50, 147.49, 132.82, 131.38, 127.39, 120.61, 114.50, 113.07, 49.15, 33.52. HRMS (ESI-TOF): calculated for $C_{14}H_{11}Cl_3N_5O$ (M+H): 370.0024, found: 370.0022.

2,2'-((piperazine-1,4-diylbis(ethane-2,1-diyl))bis(azanediyl))bis(N-(1-(3,4-dichlorophenyl)-4,5-dihydro-1H-pyrazol-3-yl)pyrimidine-5-carboxamide) (VS1337).

2-chloro-N-(1-(3,4-dichlorophenyl)-4,5-dihydro-1H-pyrazol-3-yl)pyrimidine-5-carboxamide (VS1335, 150 mg, 0.41 mmol) and 2,2'-(piperazine-1,4-diyl)bis(ethan-1-amine) (35 mg, 0.2 mmol) were dissolved in EtOH (4 mL), and DIPEA (80 µL, 0.45 mmol) was added. The reaction was heated at 70 ºC overnight. The reaction mixture was cooled to room temperature, and the precipitate was filtered, washed with EtOH and dried affording the desired product VS1337 as a yellowish solid (110 mg, 66% yield). VS1337 has limited solubility in DMSO (up to 3 mM) but is well soluble in NMP. ^{1}H NMR (400 MHz, DMSO-d6) δ 8.81 (d, *J* = 18.4 Hz, 4H), 7.76 (t, *J* = 5.8 Hz, 2H), 7.42 (d, *J* = 8.9 Hz, 2H), 7.11 (d, *J* = 2.7 Hz, 2H), 6.87 (dd, *J* = 8.9, 2.7 Hz, 2H), 3.77 (t, *J* = 9.8 Hz, 4H), 3.56 – 3.45 (m, 8H), 2.50 – 2.44 (m, 4H, overlapping with DMSO). ^{13}C NMR (101 MHz, DMSO-d6) δ 162.84, 162.77, 158.61, 150.98, 146.53, 131.36, 130.67, 118.47, 115.25, 113.26, 112.35, 56.65, 52.79, 47.97, 38.25, 32.93. HRMS (ESI-TOF): calculated for $C_{36}H_{39}Cl_4N_{14}O_2$ (M+H): 841.2100, found: 841.2099.

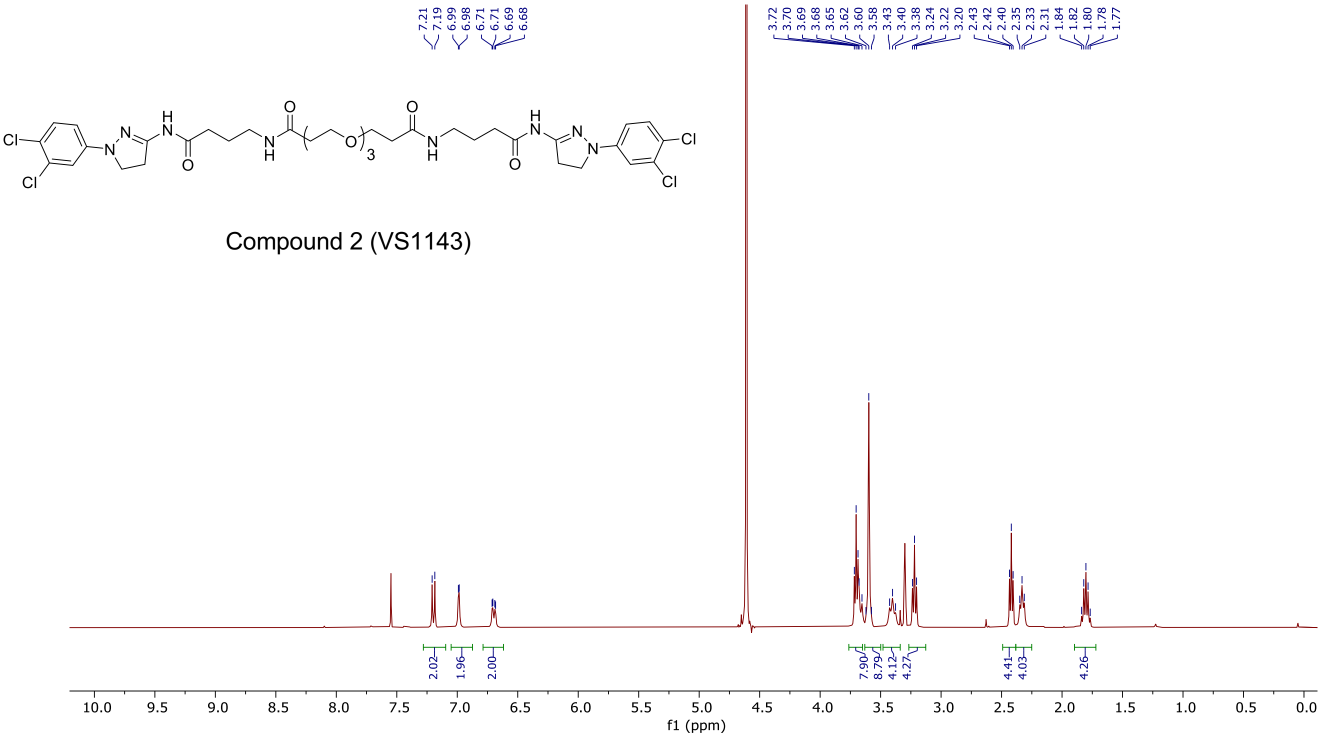
7.21
7.19
6.99
6.98
6.71
6.71
6.69
6.68
3.72
3.70
3.69
3.68
3.65
3.62
3.60
3.58
3.43
3.40
3.38
3.24
3.22
3.20
2.43
2.42
2.40
2.35
2.33
2.31
1.84
1.82
1.80
1.78
1.77
Cl
Cl
N
N
H
N
O
N
H
O
O
3
O
N
H
O
H
N
N
N
Cl
Cl
Compound 2 (VS1143)
2.02
1.96
2.00
7.90
8.79
4.12
4.27
4.41
4.03
4.26
10.0 9.5 9.0 8.5 8.0 7.5 7.0 6.5 6.0 5.5 5.0 4.5 4.0 3.5 3.0 2.5 2.0 1.5 1.0 0.5 0.0
f1 (ppm)

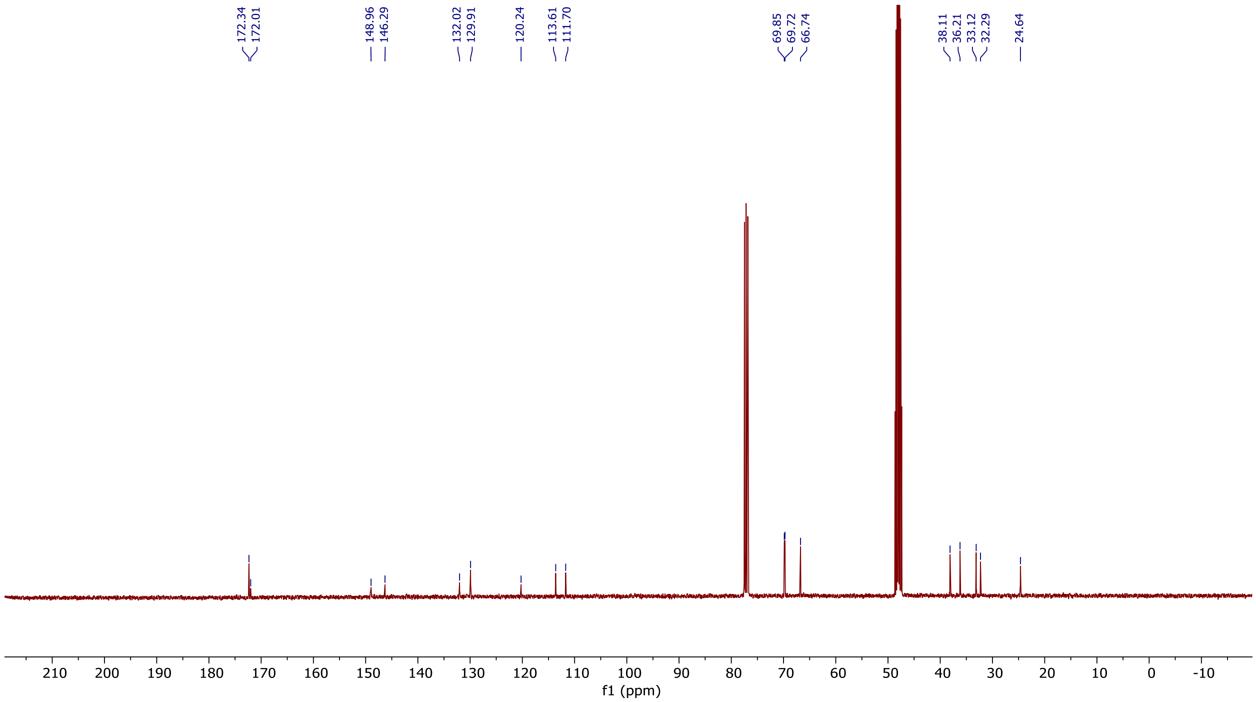
172.34
172.01
148.96
146.29
132.02
129.91
120.24
113.61
111.70
69.85
69.72
66.74
38.11
36.21
33.12
32.29
24.64
210 200 190 180 170 160 150 140 130 120 110 100 90 80 70 60 50 40 30 20 10 0 -10
f1 (ppm)

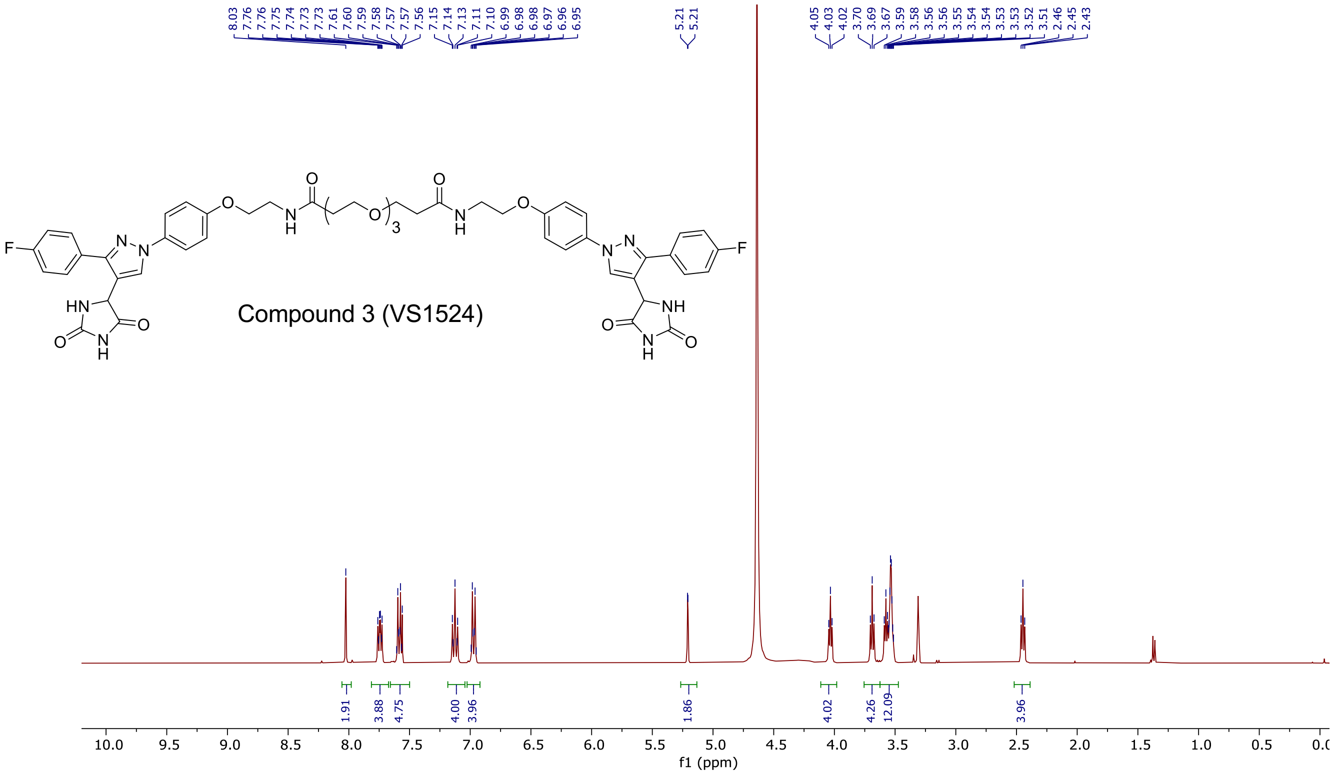
8.03 7.76 7.76 7.75 7.74 7.73 7.61 7.60 7.59 7.58 7.57 7.57 7.57 7.56 7.15 7.14 7.13 7.11 7.10 6.99 6.98 6.98 6.97 6.96 6.95
5.21 5.21
4.05 4.03 4.02 3.70 3.69 3.67 3.59 3.58 3.56 3.56 3.55 3.54 3.54 3.53 3.53 3.52 3.51 2.46 2.45 2.43
Compound 3 (VS1524)
1.91 3.88 4.75 4.00 3.96 1.86 4.02 4.26 12.09 3.96
f1 (ppm)

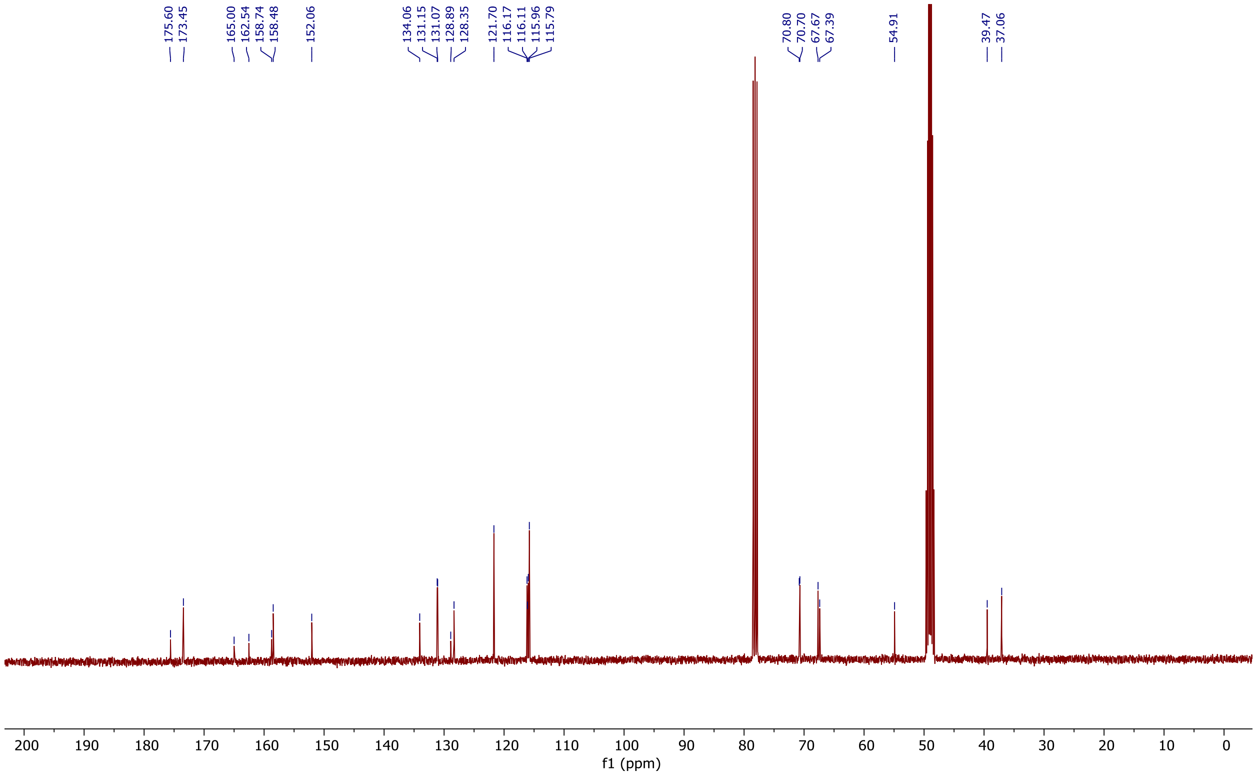
175.60 173.45 165.00 162.54 158.74 158.48 152.06 134.06 131.15 131.07 128.89 128.35 121.70 116.17 116.11 115.96 115.79 70.80 70.70 67.67 67.39 54.91 39.47 37.06
f1 (ppm)

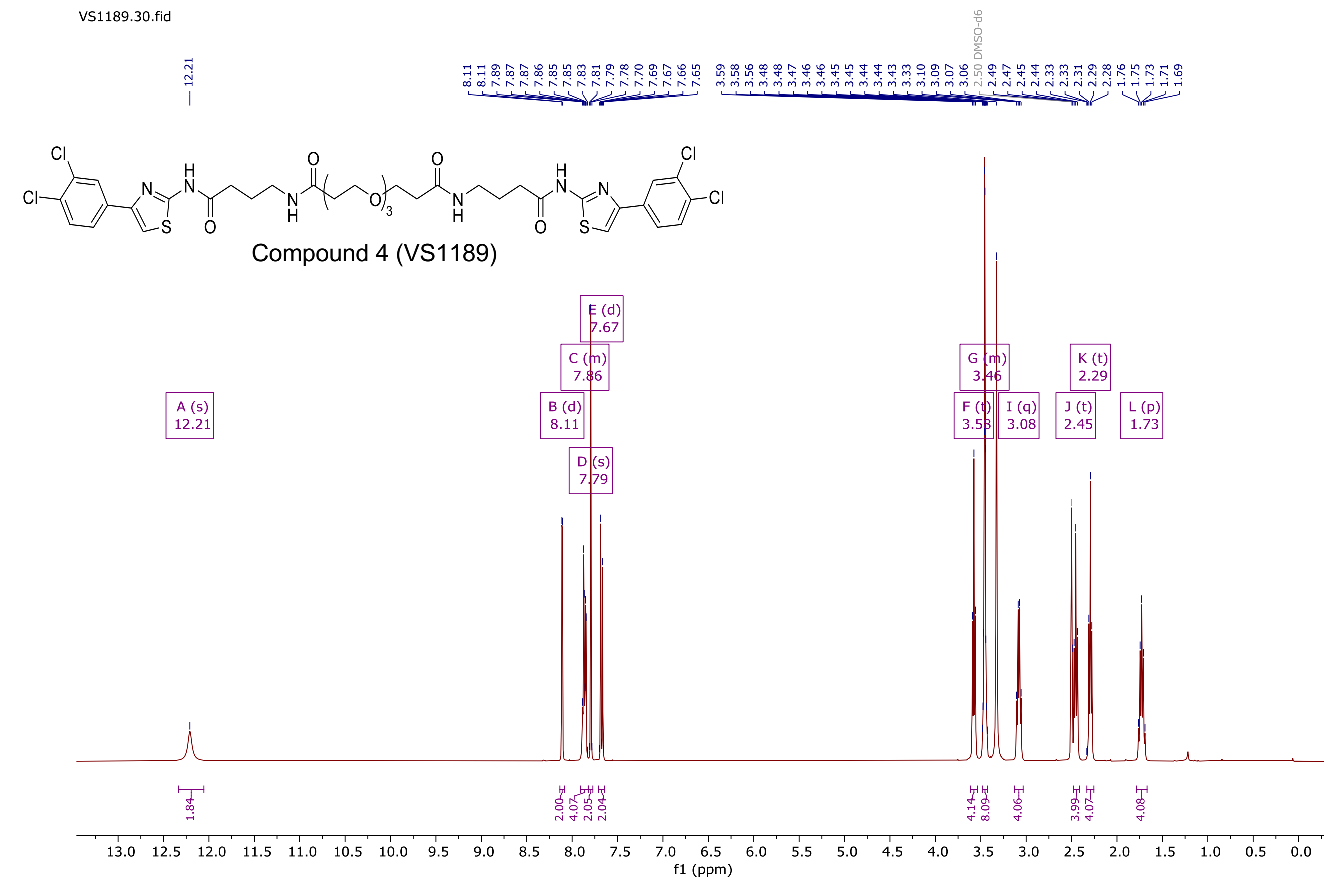

Compound 4 (VS1189)

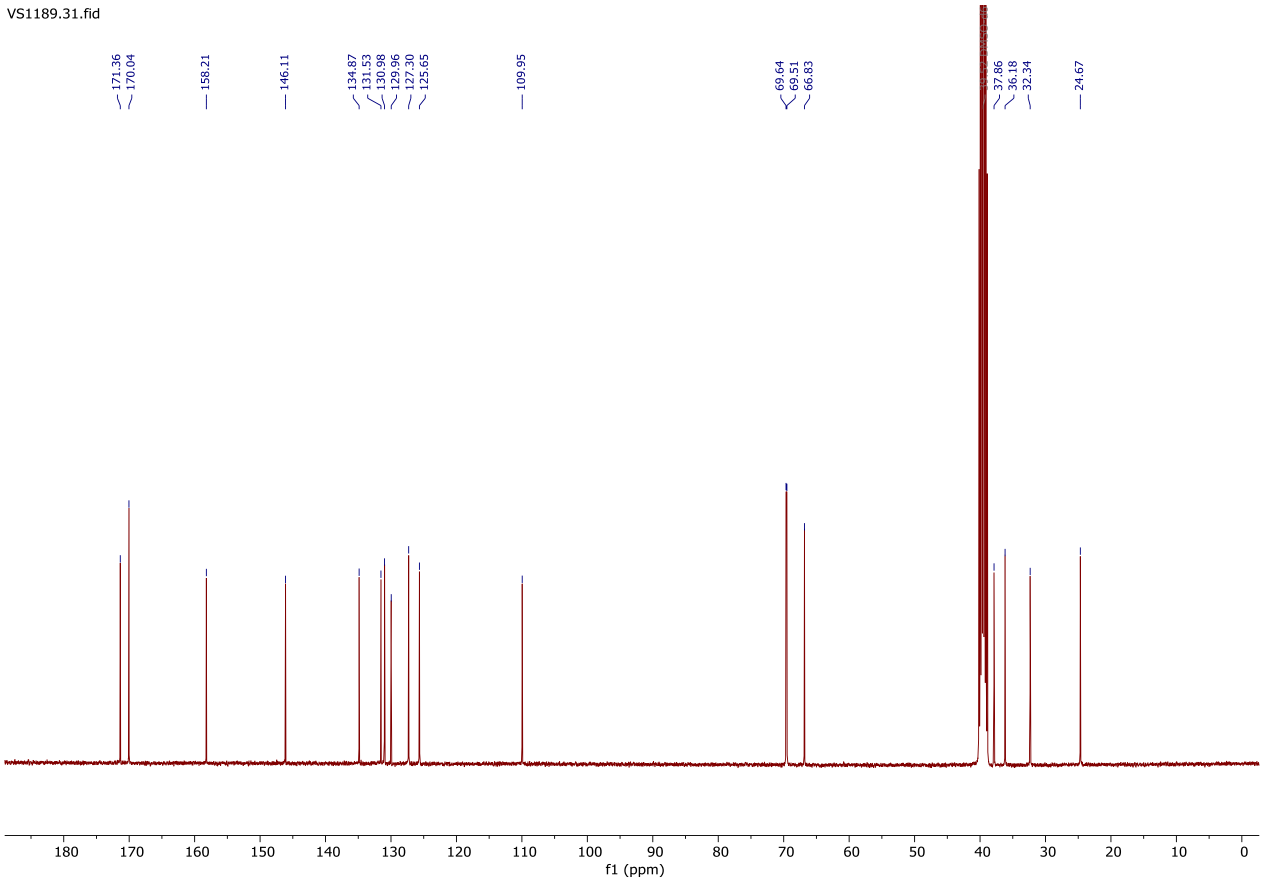

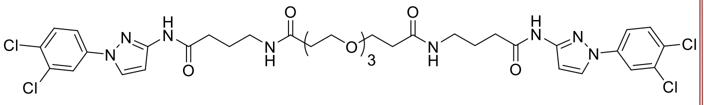

Compound 5 (VS1144)

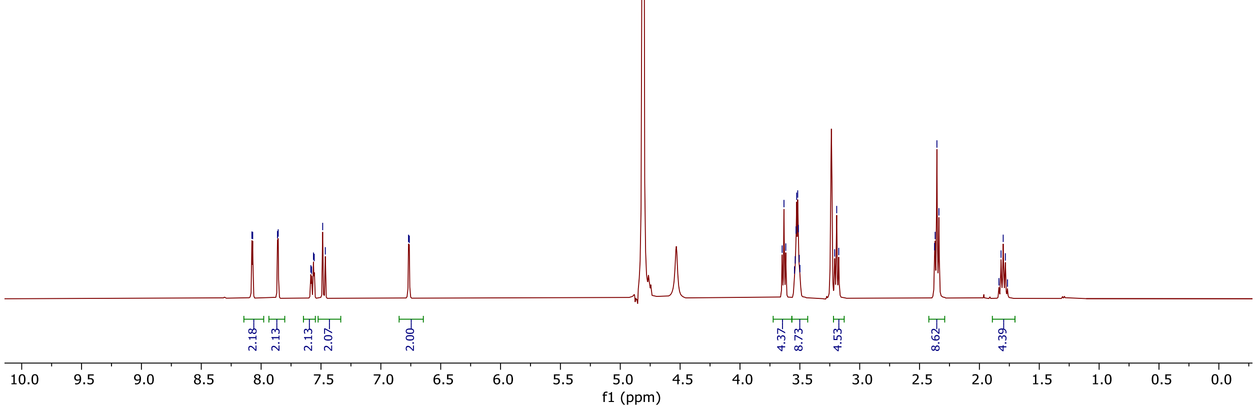

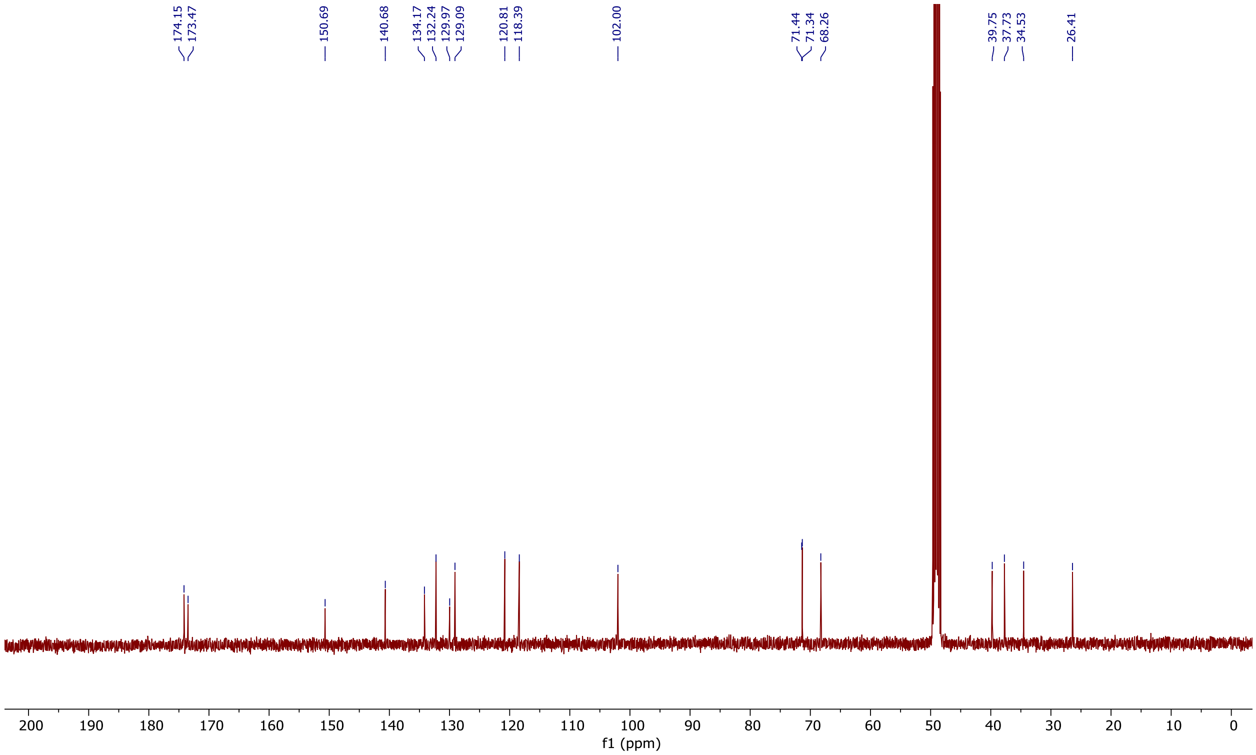

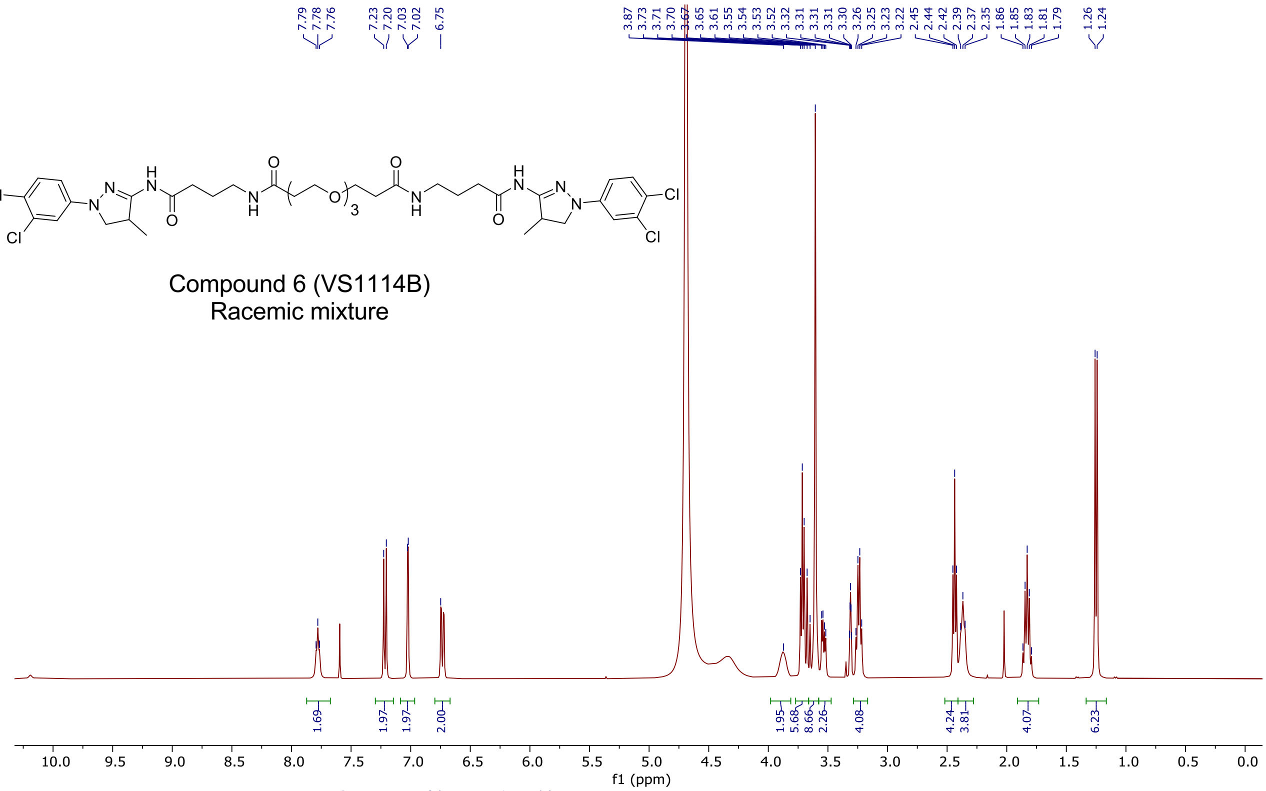
Compound 6 (VS1114B)
Racemic mixture
f1 (ppm)

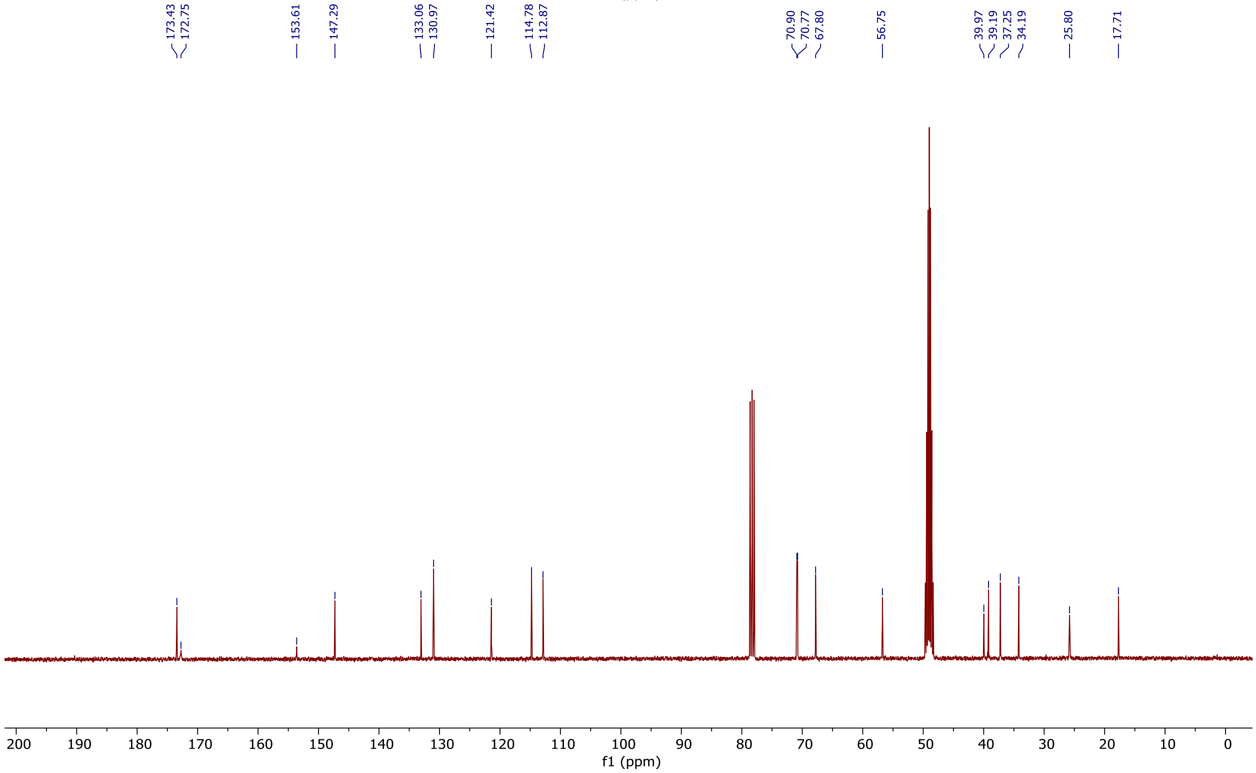
f1 (ppm)

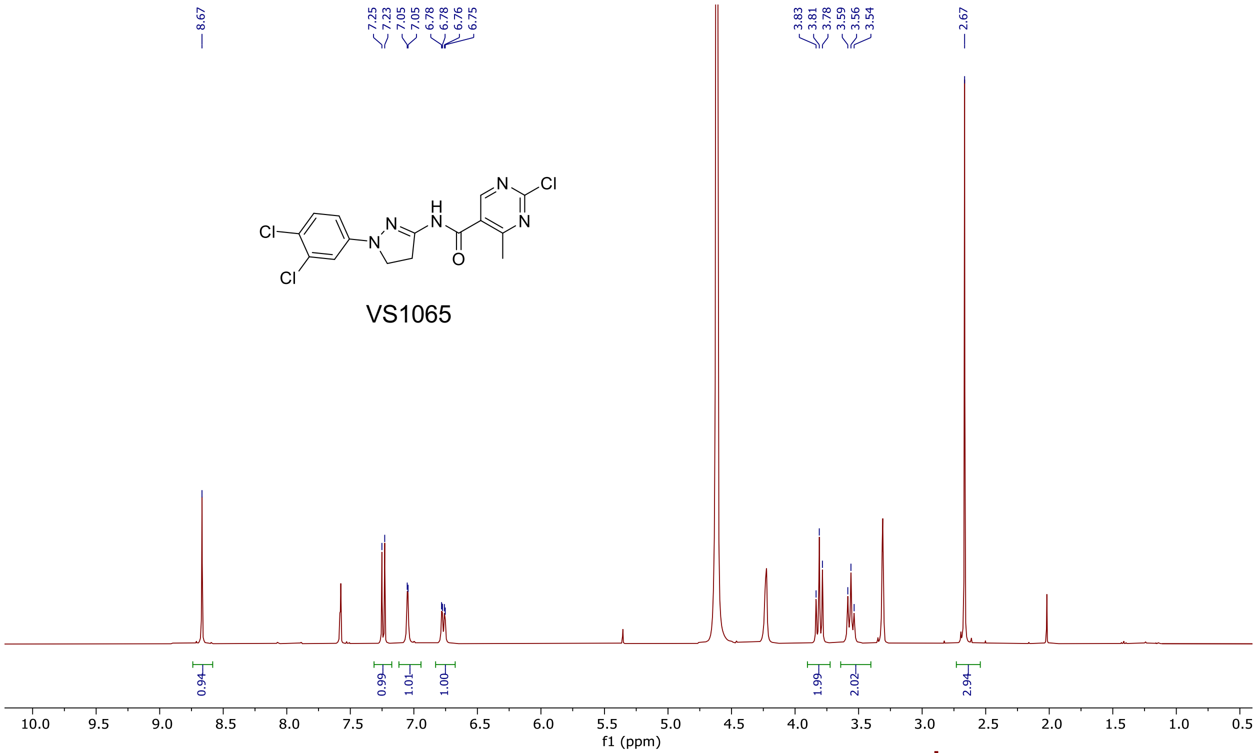
8.67
7.25
7.23
7.05
7.05
6.78
6.78
6.76
6.75
3.83
3.81
3.78
3.59
3.56
3.54
2.67
Cl
Cl
N
N
H
N
O
N
N
Cl
VS1065
0.94
0.99
1.01
1.00
1.99
2.02
2.94
10.0
9.5
9.0
8.5
8.0
7.5
7.0
6.5
6.0
5.5
5.0
4.5
4.0
3.5
3.0
2.5
2.0
1.5
1.0
0.5
f1 (ppm)

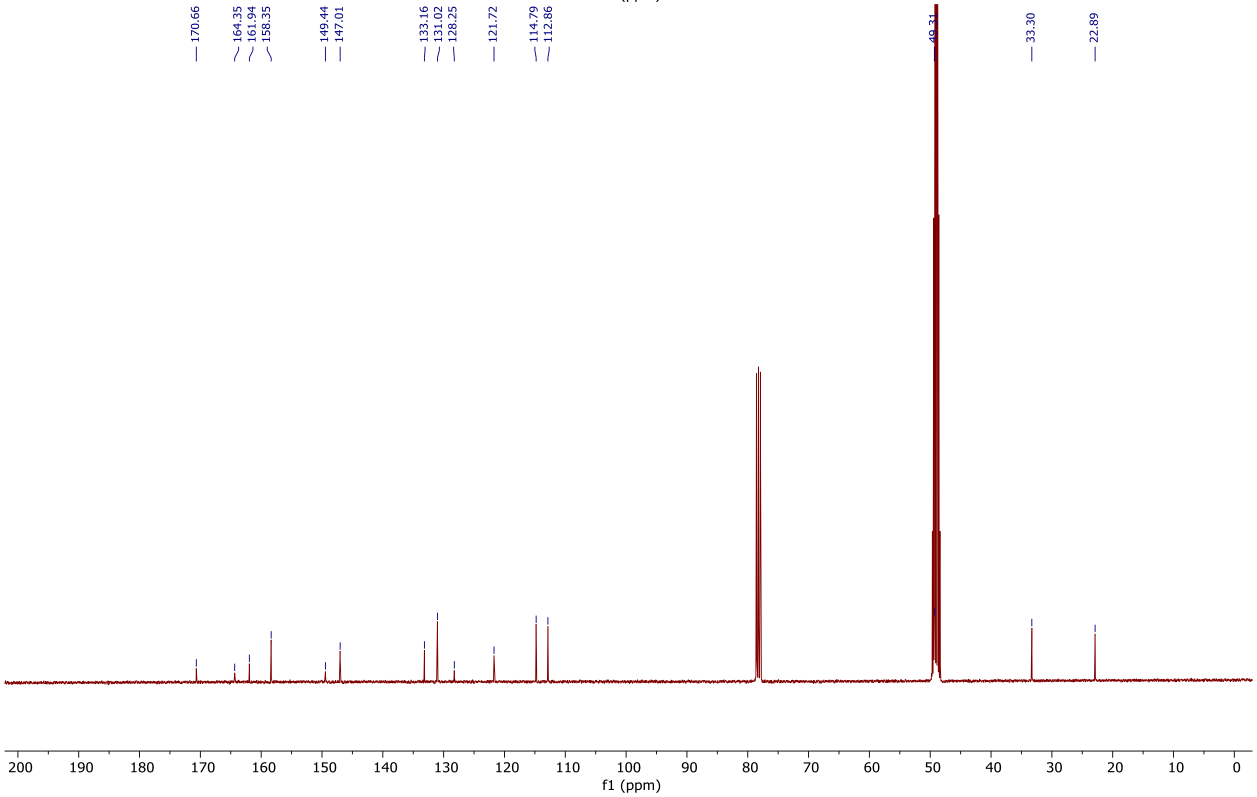
170.66
164.35
161.94
158.35
149.44
147.01
133.16
131.02
128.25
121.72
114.79
112.86
49.31
33.30
22.89
200
190
180
170
160
150
140
130
120
110
100
90
80
70
60
50
40
30
20
10
0
f1 (ppm)

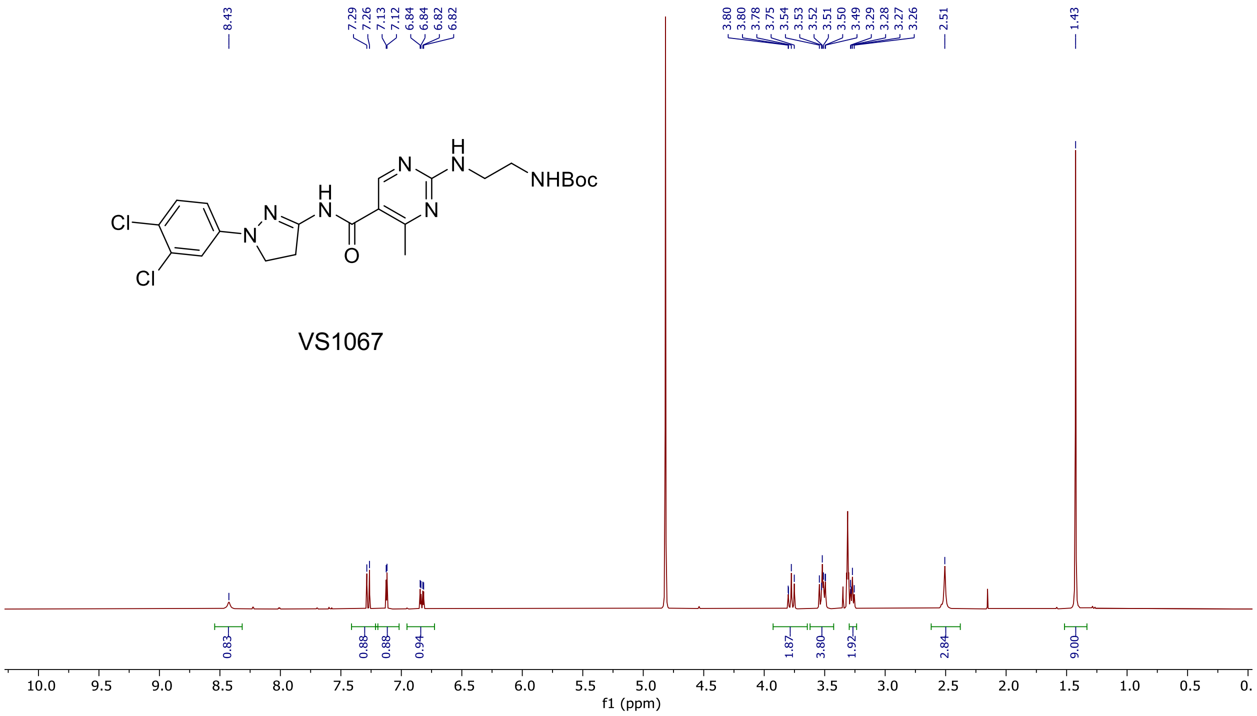
8.43
7.29
7.26
7.13
7.12
6.84
6.84
6.82
6.82
3.80
3.80
3.78
3.75
3.54
3.53
3.52
3.51
3.50
3.49
3.29
3.28
3.27
3.26
2.51
1.43
Cl
Cl
N
N
H
N
O
N
N
H
N
NHBoc
VS1067
0.83
0.88
0.88
0.94
1.87
3.80
1.92
2.84
9.00
10.0 9.5 9.0 8.5 8.0 7.5 7.0 6.5 6.0 5.5 5.0 4.5 4.0 3.5 3.0 2.5 2.0 1.5 1.0 0.5 0.
f1 (ppm)

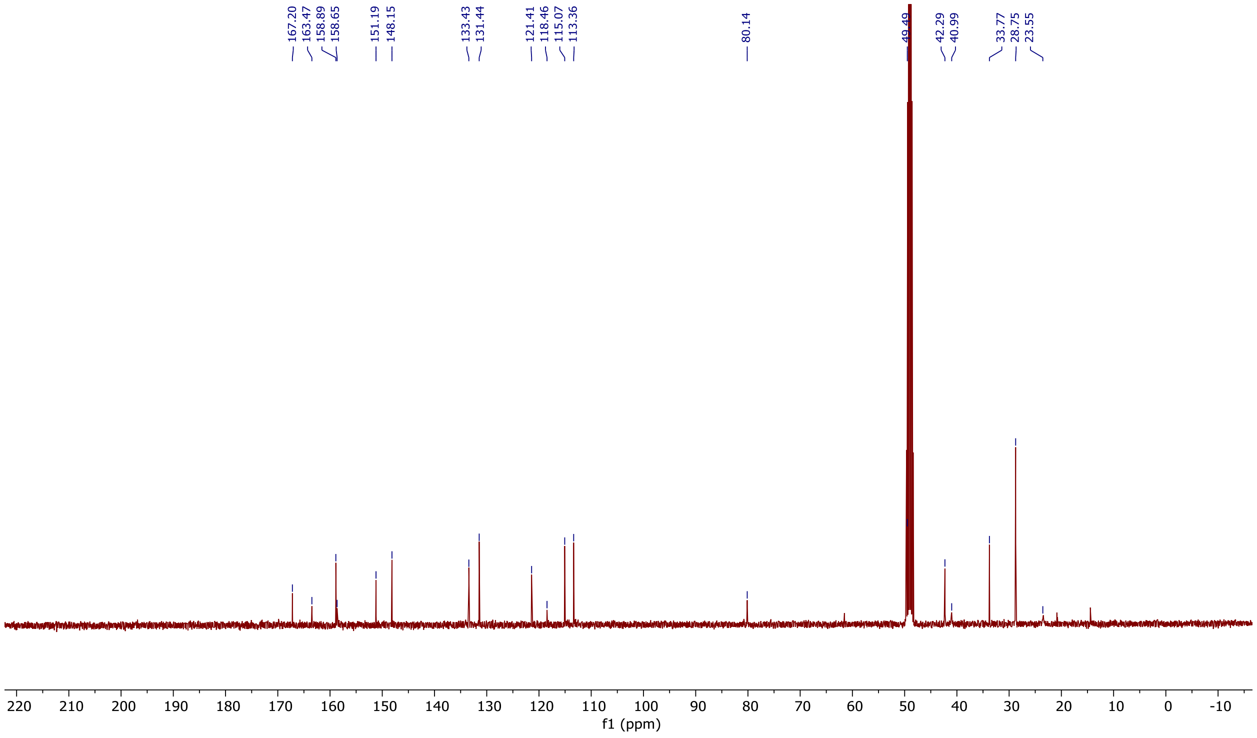
167.20
163.47
158.89
158.65
151.19
148.15
133.43
131.44
121.41
118.46
115.07
113.36
80.14
49.49
42.29
40.99
33.77
28.75
23.55
220 210 200 190 180 170 160 150 140 130 120 110 100 90 80 70 60 50 40 30 20 10 0 -10
f1 (ppm)

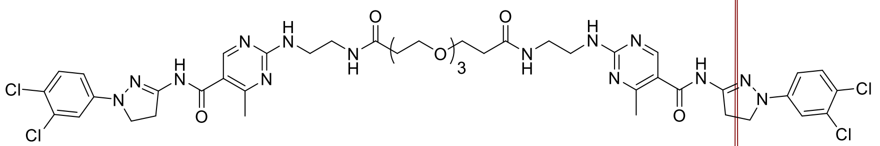

Compound 9 (VS1142)

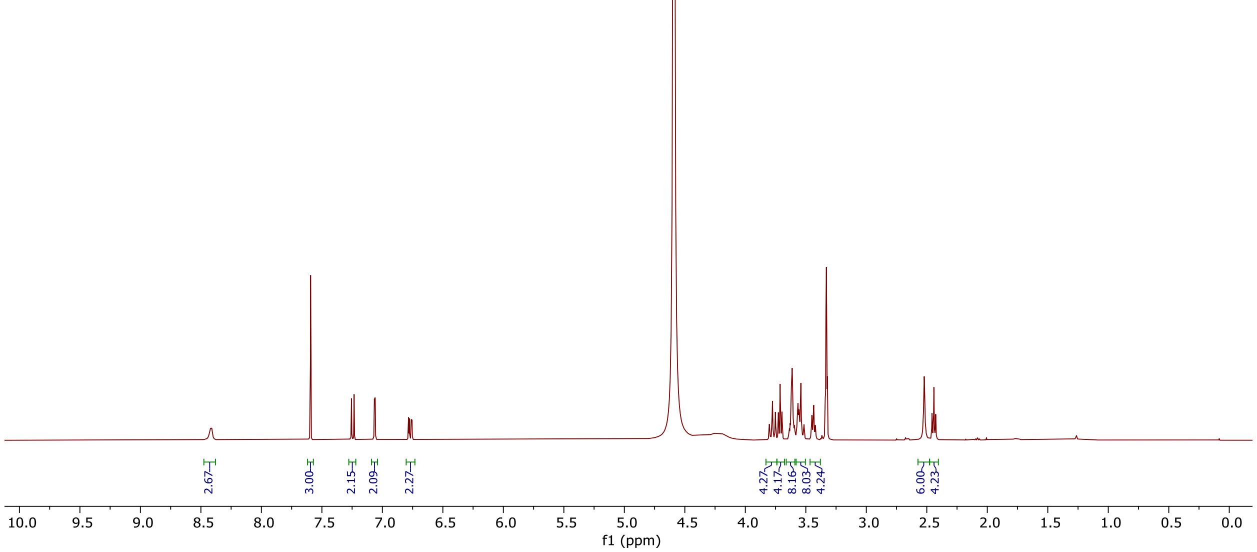

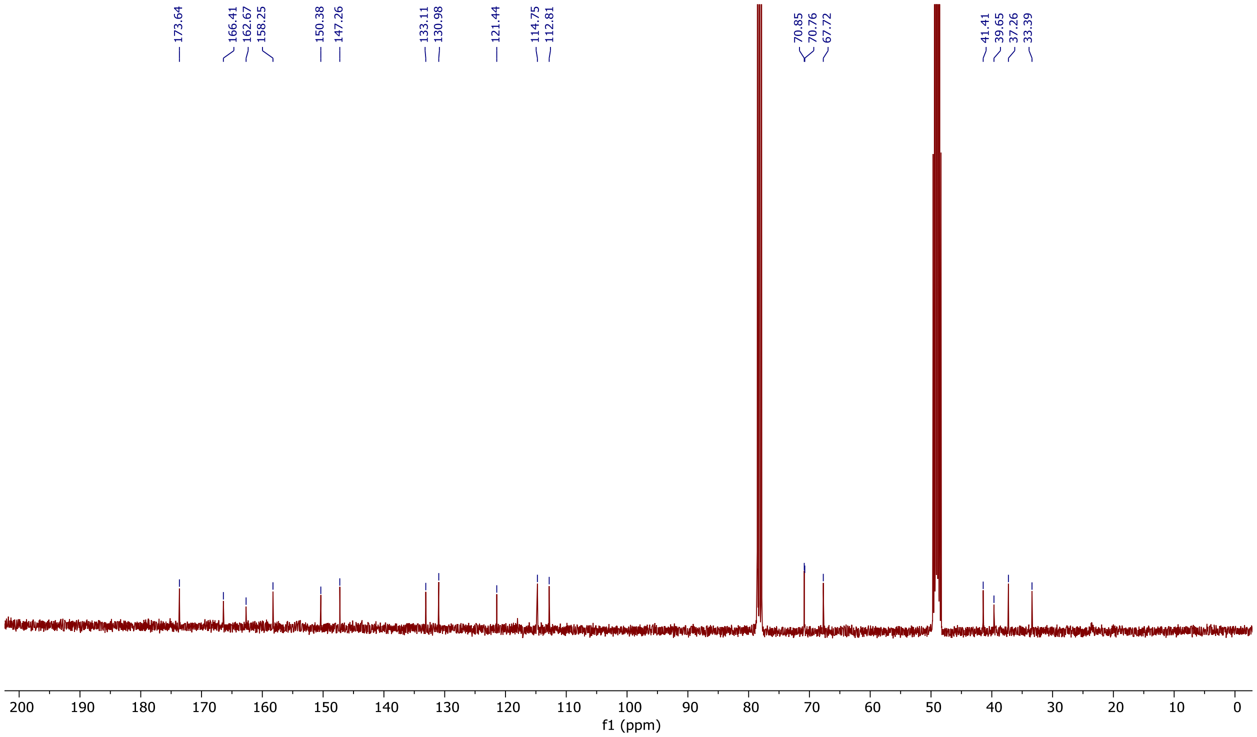

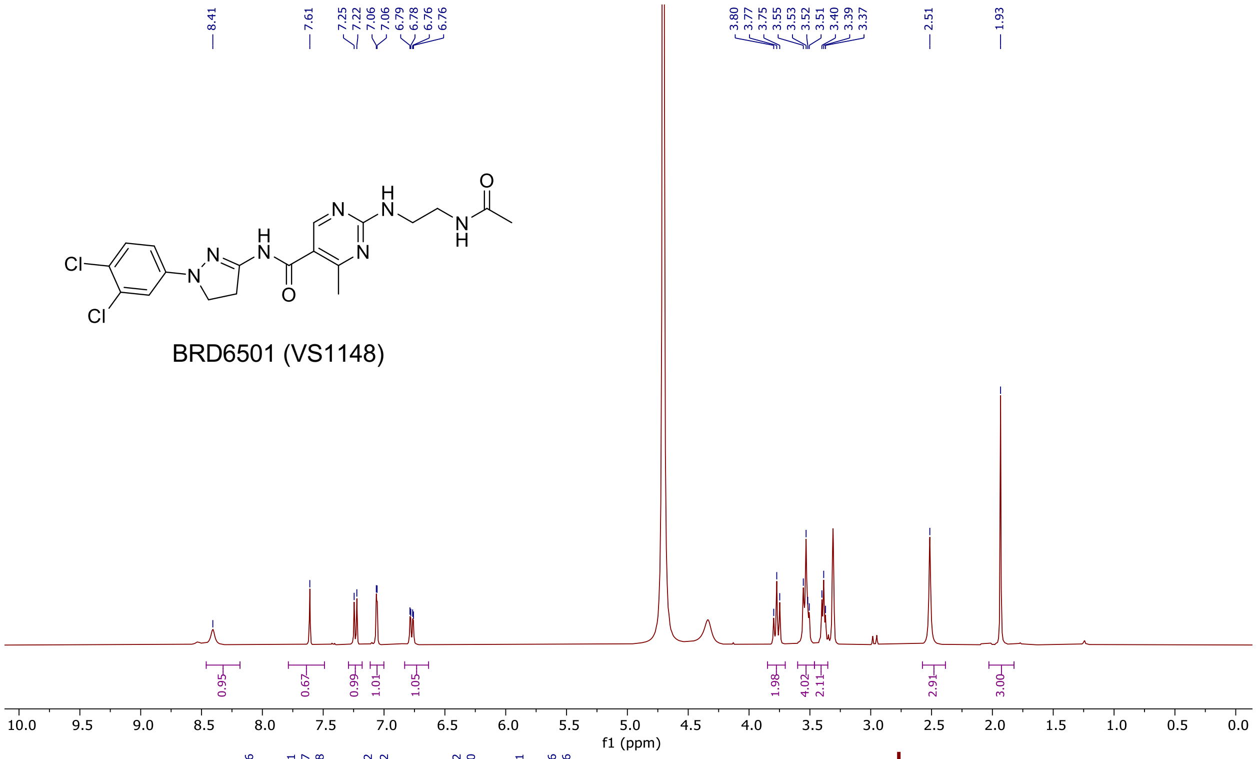
BRD6501 (VS1148)
8.41
7.61
7.25 7.22 7.06 7.06 6.79 6.78 6.76 6.76
3.80 3.77 3.75 3.55 3.53 3.52 3.51 3.40 3.39 3.37
2.51
1.93
0.95 0.67 0.99 1.01 1.05 1.98 4.02 2.11 2.91 3.00
f1 (ppm)

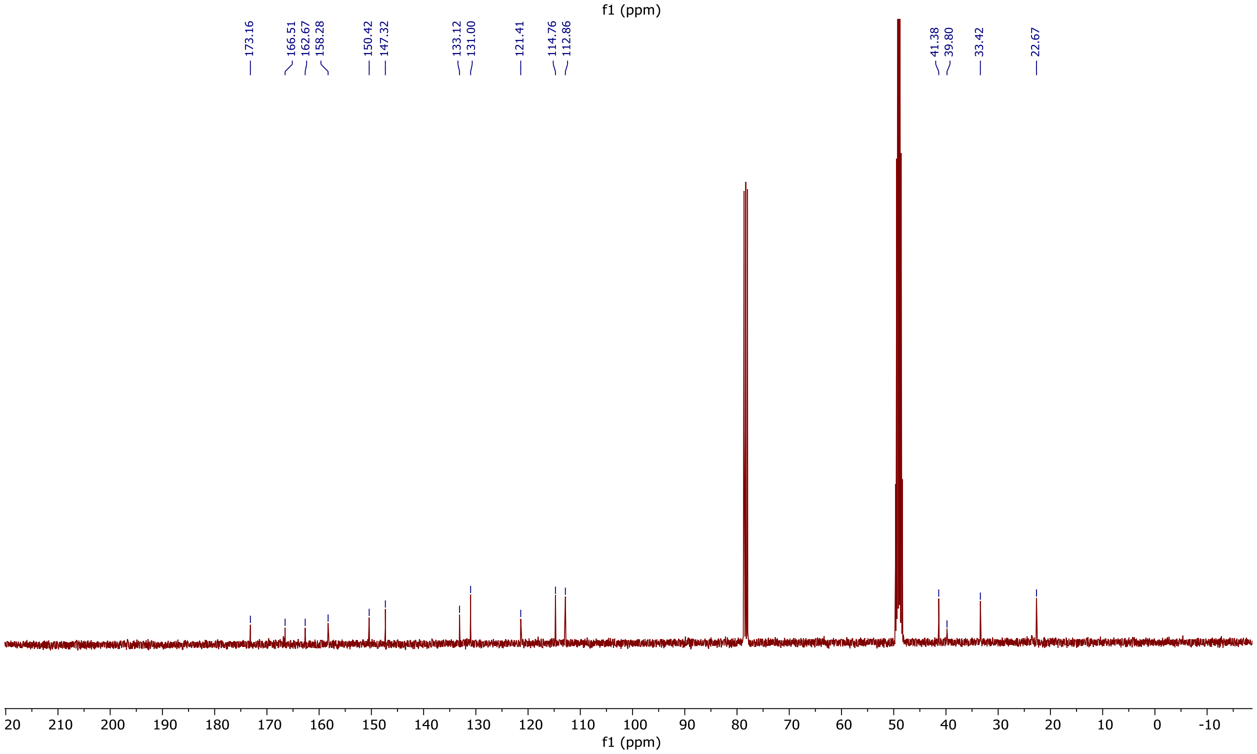
173.16
166.51 162.67 158.28
150.42 147.32
133.12 131.00
121.41
114.76 112.86
41.38 39.80
33.42
22.67
f1 (ppm)

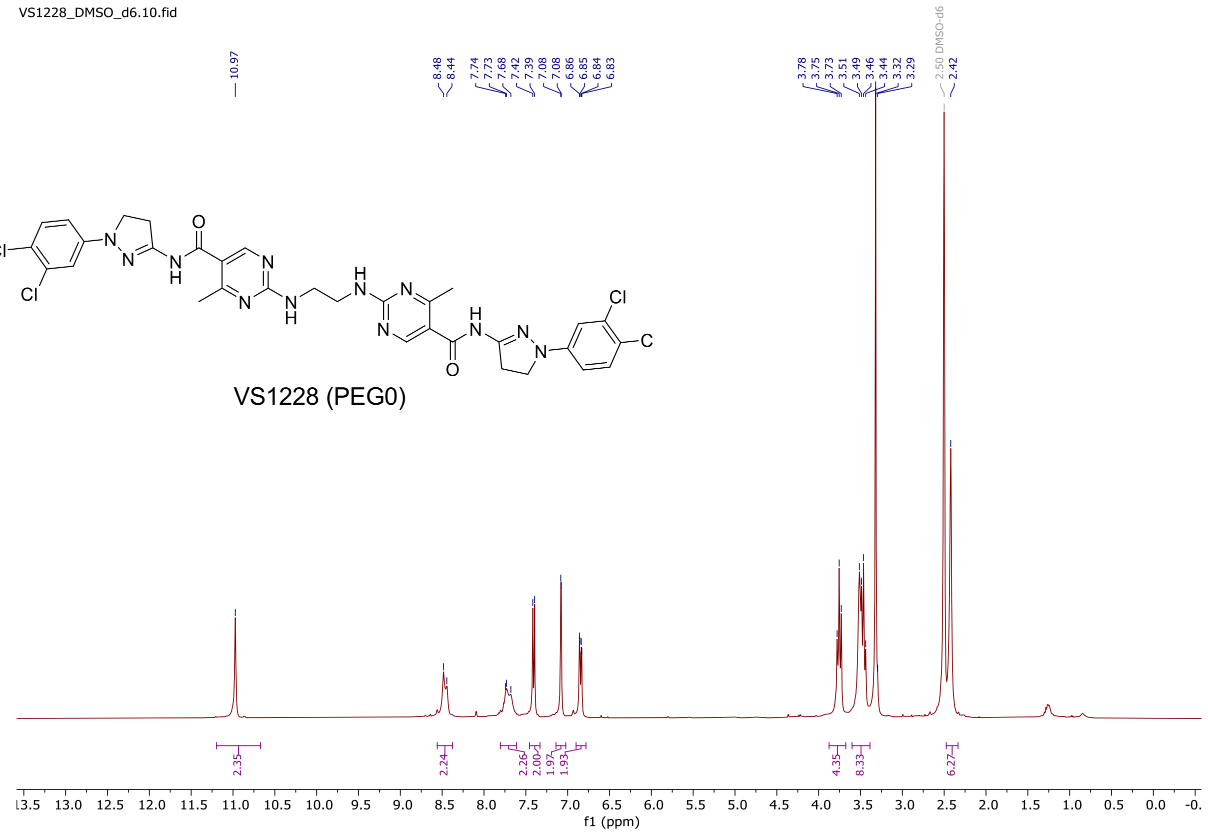
VS1228_DMSO_d6.10.fid
10.97
8.48
8.44
7.74
7.73
7.68
7.42
7.39
7.08
7.08
6.86
6.85
6.84
6.83
3.78
3.75
3.73
3.51
3.49
3.46
3.44
3.32
3.29
2.50 DMSO-d6
2.42
VS1228 (PEG0)
2.35
2.24
2.26
2.00
1.97
1.93
4.35
8.33
6.27
f1 (ppm)

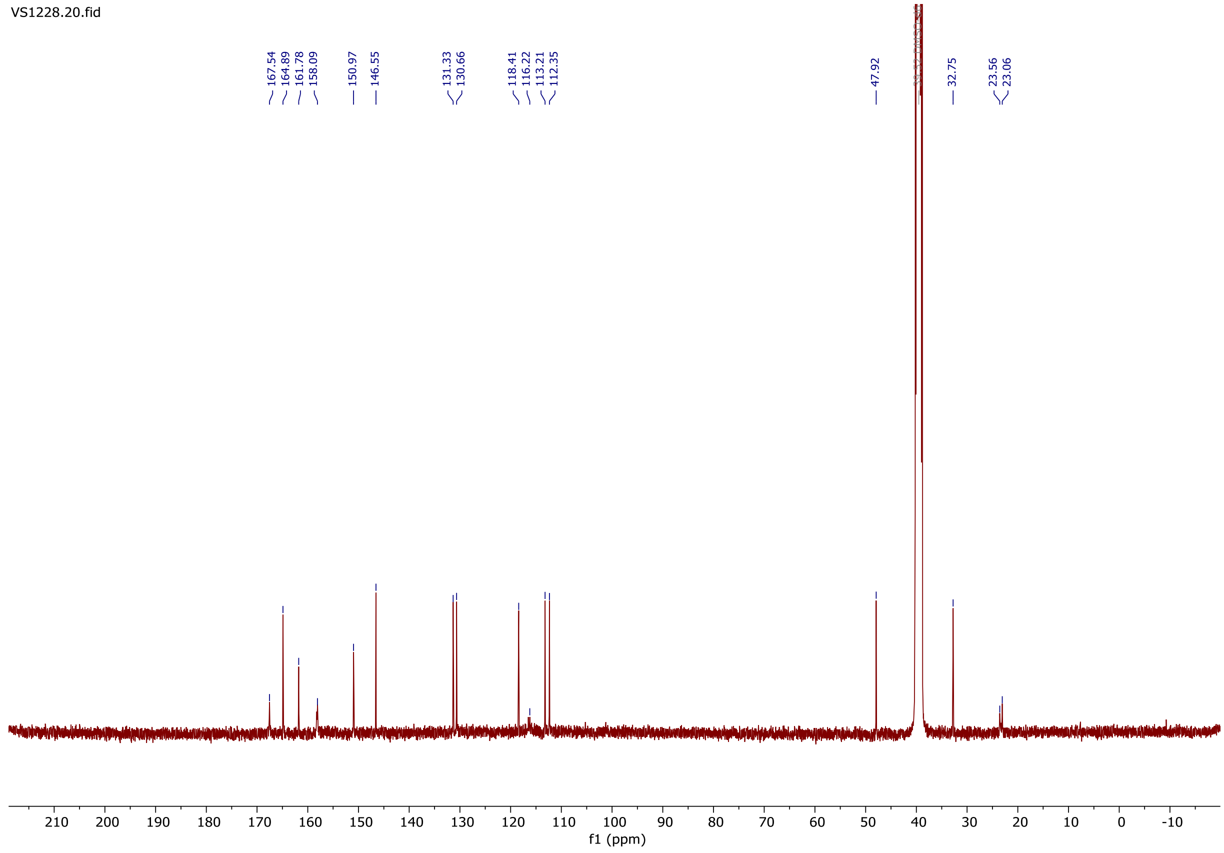
VS1228.20.fid
167.54
164.89
161.78
158.09
150.97
146.55
131.33
130.66
118.41
116.22
113.21
112.35
47.92
32.75
23.56
23.06
f1 (ppm)

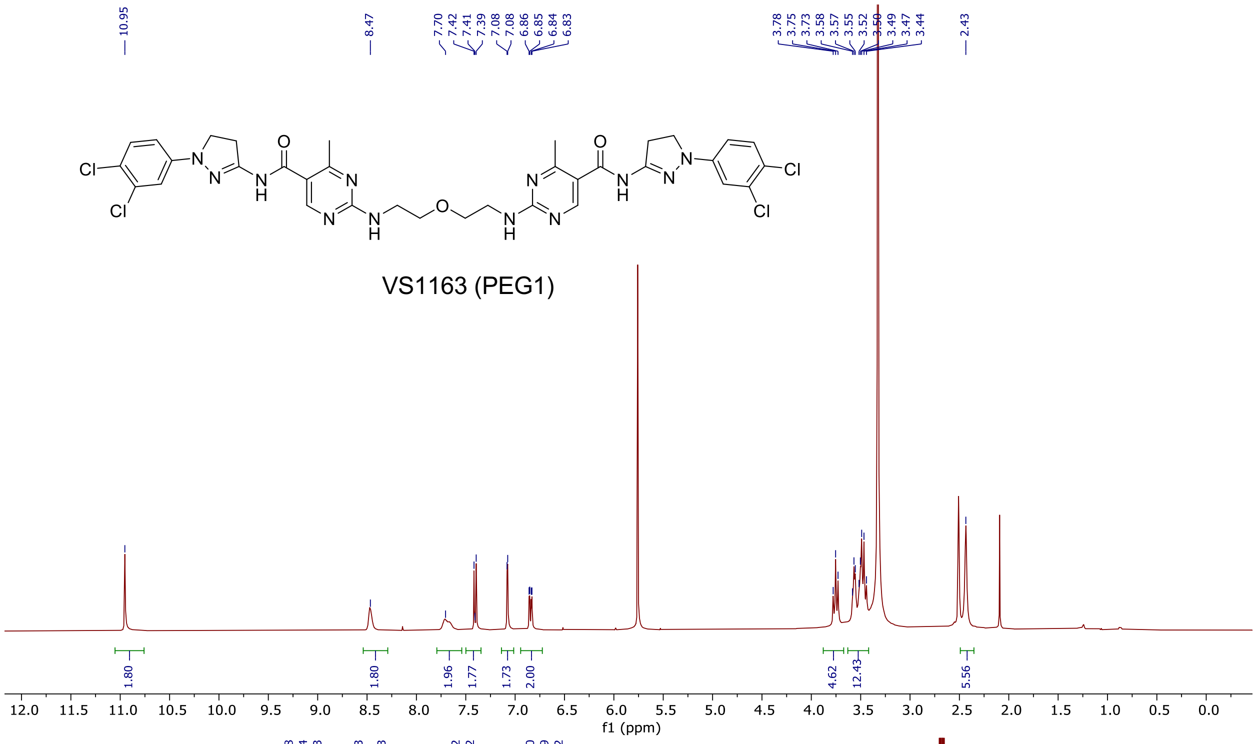
VS1163 (PEG1)
f1 (ppm)

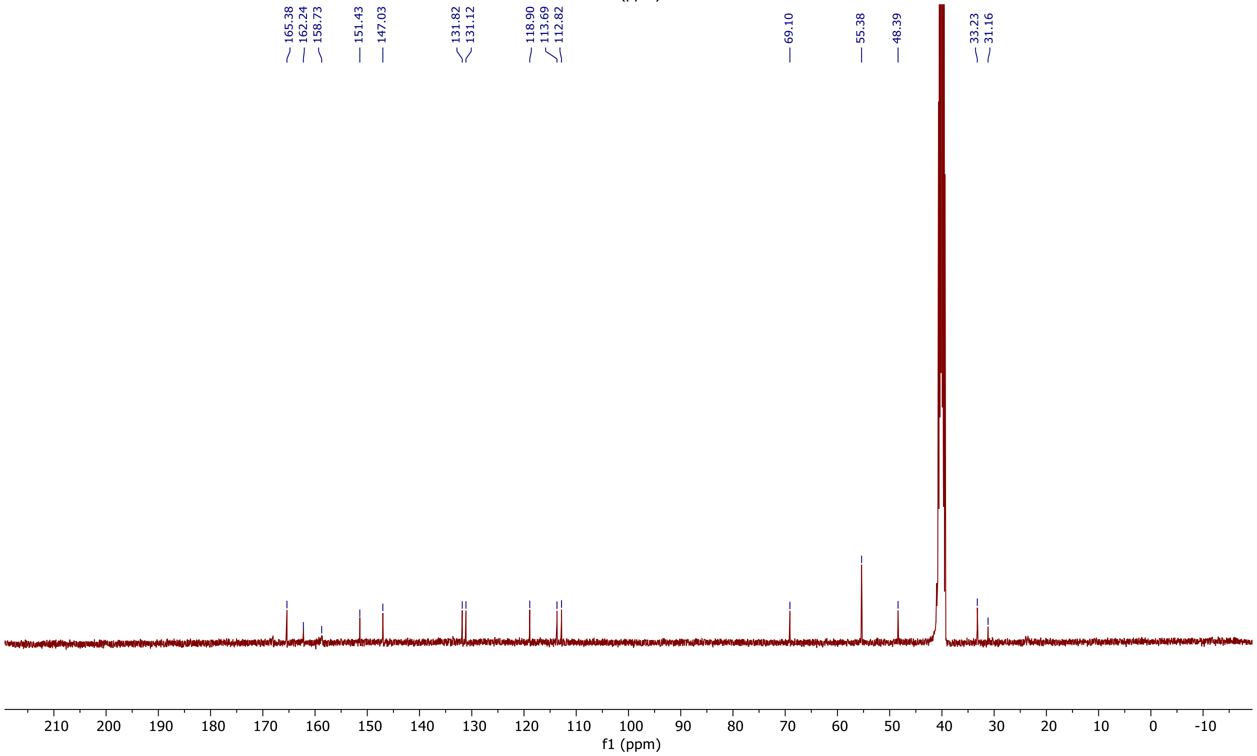
f1 (ppm)

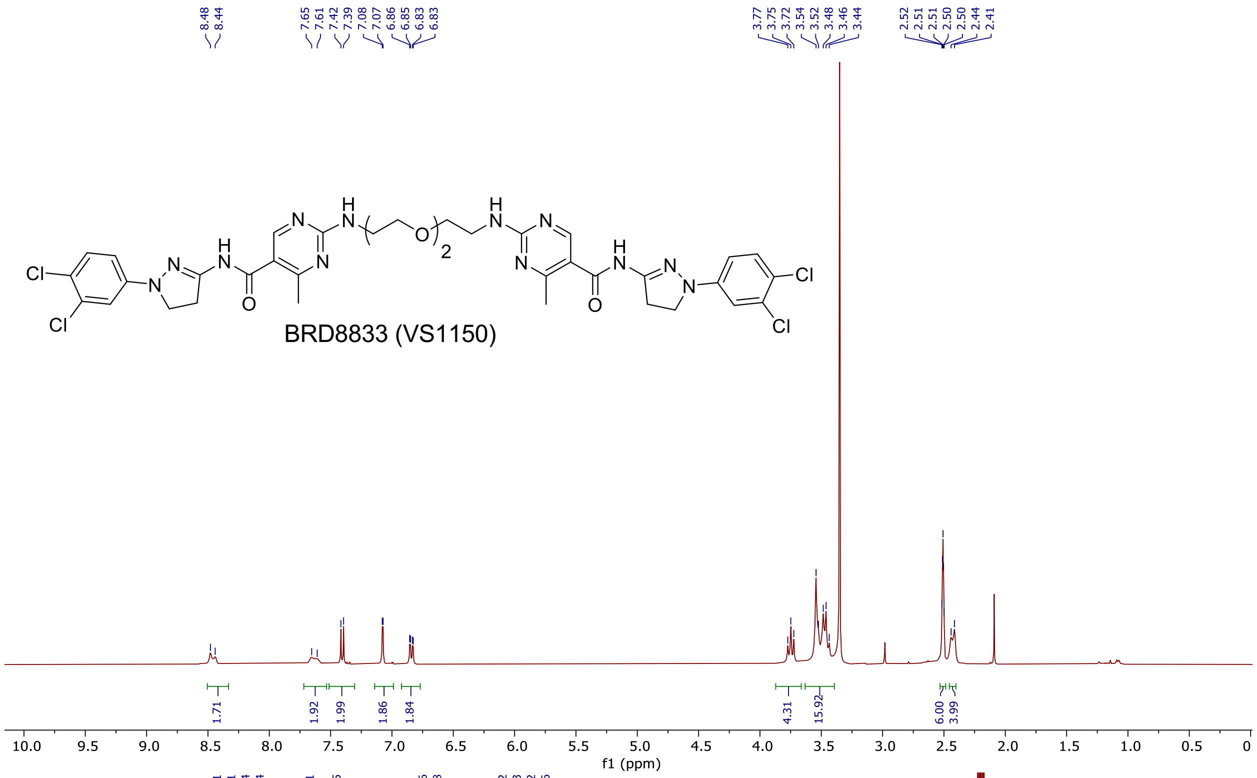
8.48
8.44
7.65
7.61
7.42
7.39
7.08
7.07
6.86
6.85
6.83
6.83
3.77
3.75
3.72
3.54
3.52
3.48
3.46
3.44
2.52
2.51
2.51
2.50
2.50
2.44
2.41
BRD8833 (VS1150)
1.71
1.92
1.99
1.86
1.84
4.31
15.92
6.00
3.99
f1 (ppm)

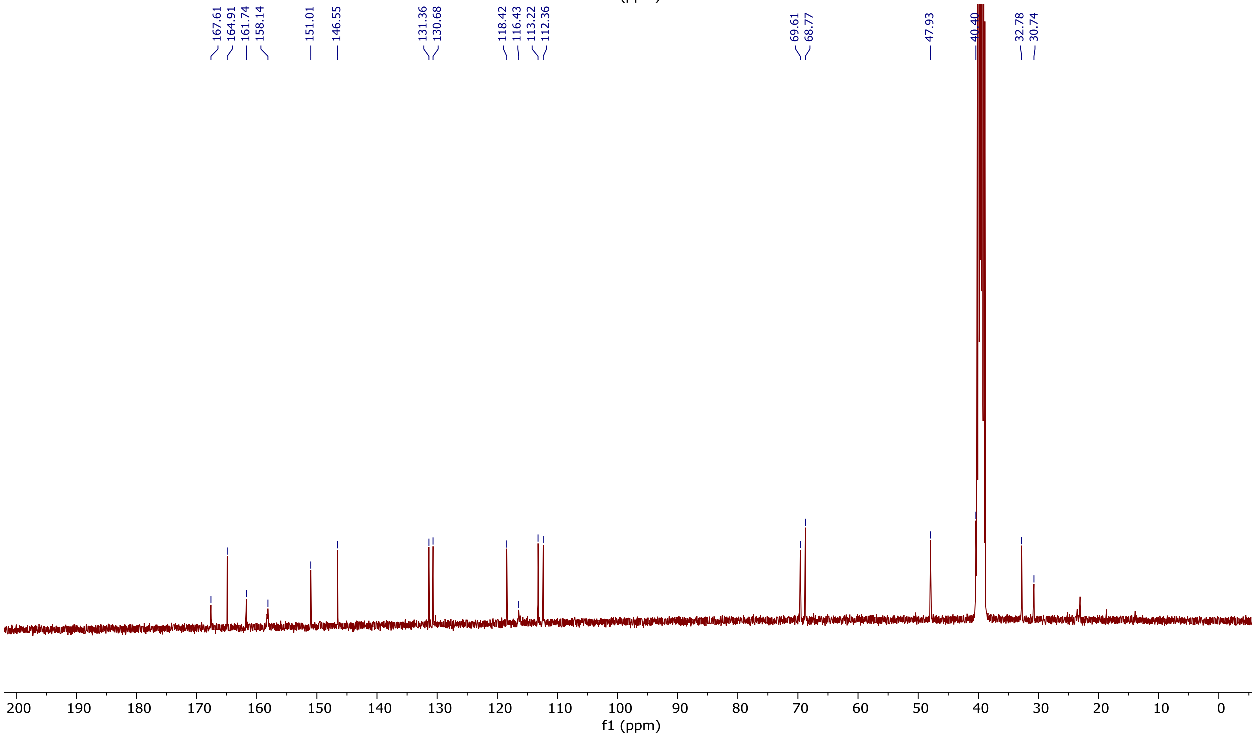
167.61
164.91
161.74
158.14
151.01
146.55
131.36
130.68
118.42
116.43
113.22
112.36
69.61
68.77
47.93
40.40
32.78
30.74
f1 (ppm)

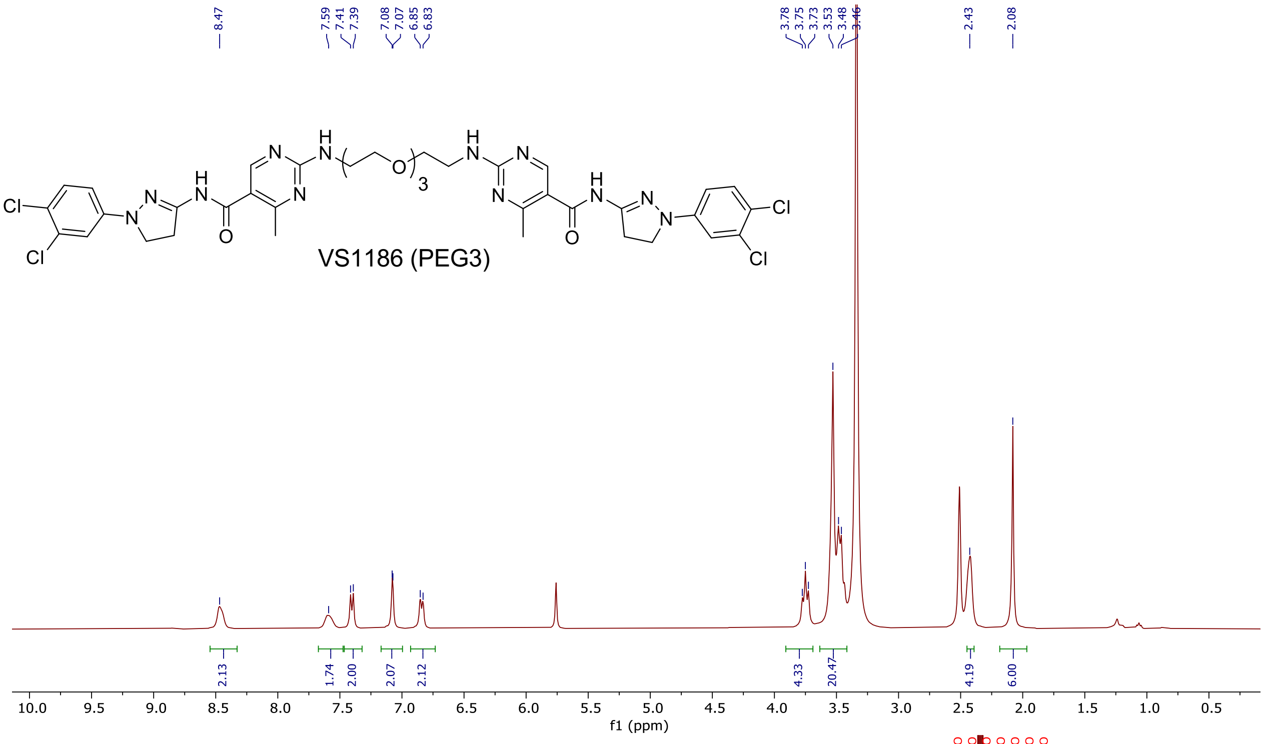
8.47
7.59
7.41
7.39
7.08
7.07
6.85
6.83
3.78
3.75
3.73
3.53
3.48
3.46
2.43
2.08
Cl
Cl
N
N
H
N
O
N
N
H
N
O
3
H
N
N
N
H
N
O
N
N
Cl
Cl
VS1186 (PEG3)
2.13
1.74
2.00
2.07
2.12
4.33
20.47
4.19
6.00
10.0
9.5
9.0
8.5
8.0
7.5
7.0
6.5
6.0
5.5
5.0
4.5
4.0
3.5
3.0
2.5
2.0
1.5
1.0
0.5
f1 (ppm)

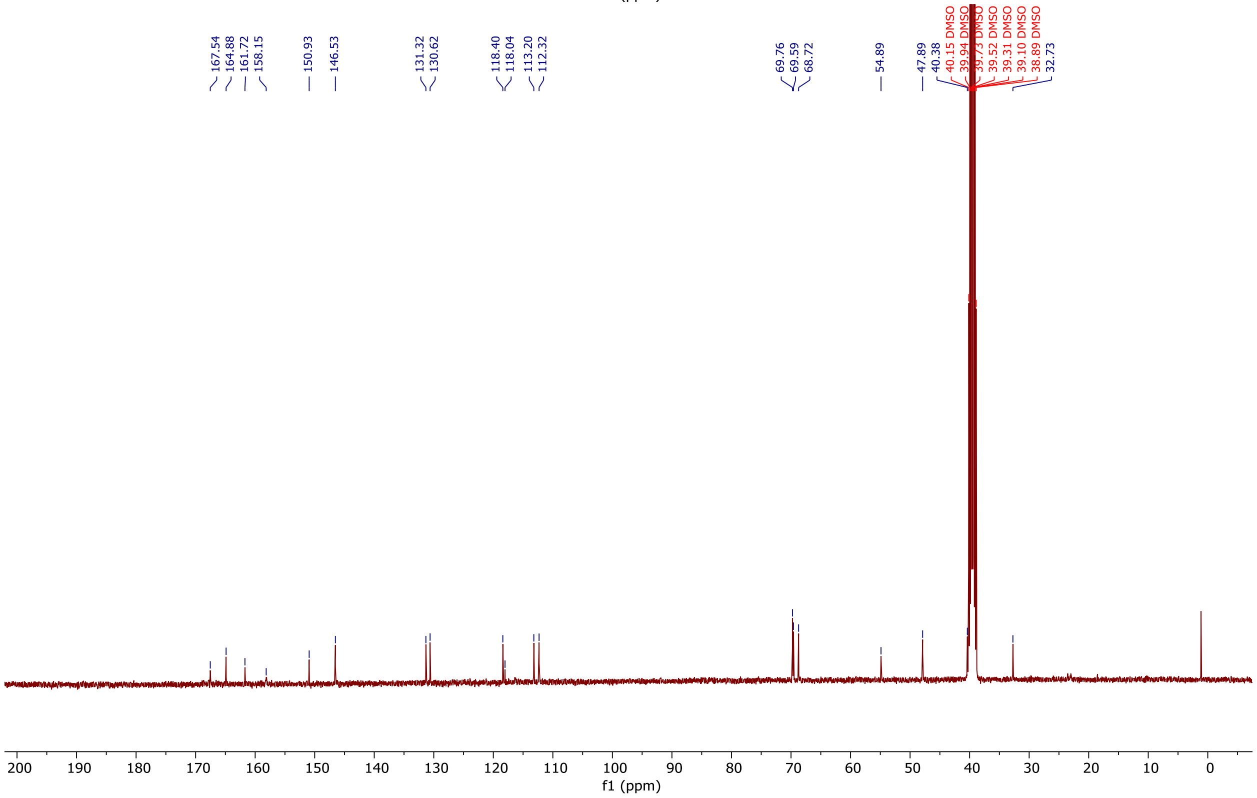
167.54
164.88
161.72
158.15
150.93
146.53
131.32
130.62
118.40
118.04
113.20
112.32
69.76
69.59
68.72
54.89
47.89
40.38
40.15 DMSO
39.94 DMSO
39.73 DMSO
39.52 DMSO
39.31 DMSO
39.10 DMSO
38.89 DMSO
32.73
200
190
180
170
160
150
140
130
120
110
100
90
80
70
60
50
40
30
20
10
0
f1 (ppm)

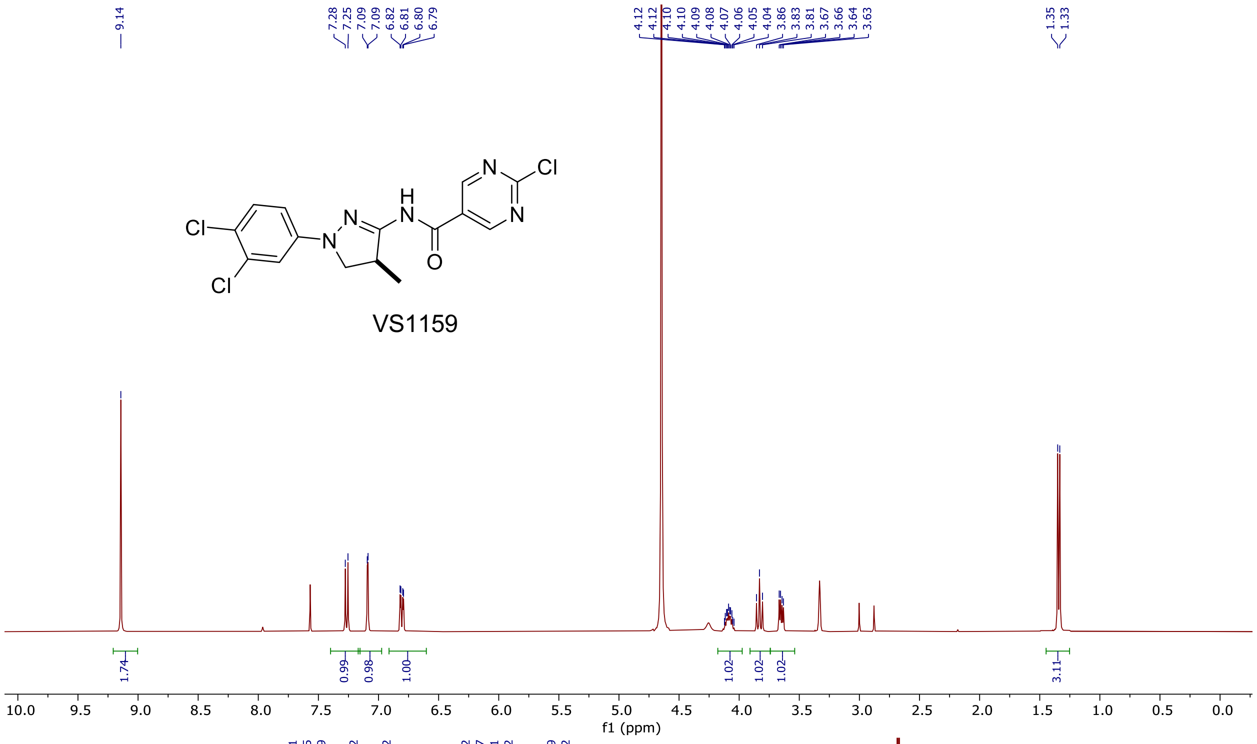
9.14
7.28
7.25
7.09
7.09
6.82
6.81
6.80
6.79
4.12
4.12
4.10
4.10
4.09
4.08
4.07
4.06
4.05
4.04
3.86
3.83
3.81
3.67
3.66
3.64
3.63
1.35
1.33
1.74
0.99
0.98
1.00
1.02
1.02
1.02
3.11
VS1159
f1 (ppm)

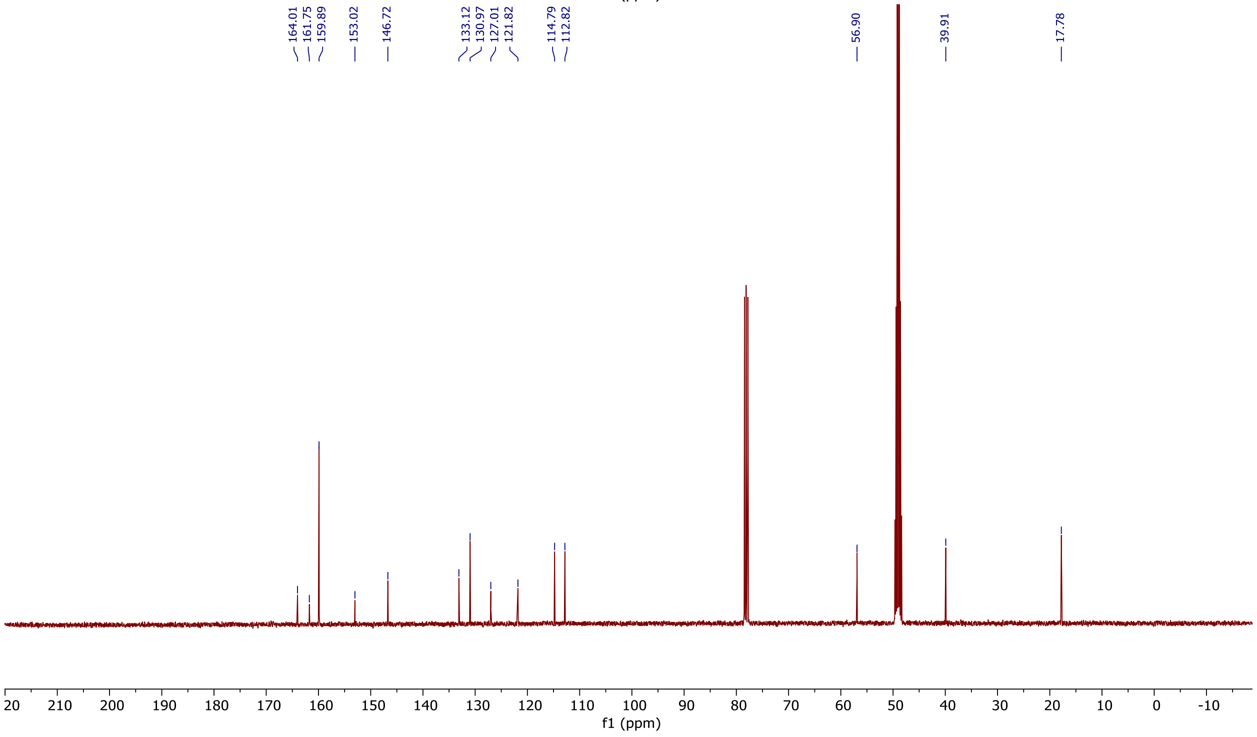
164.01
161.75
159.89
153.02
146.72
133.12
130.97
127.01
121.82
114.79
112.82
56.90
39.91
17.78
f1 (ppm)

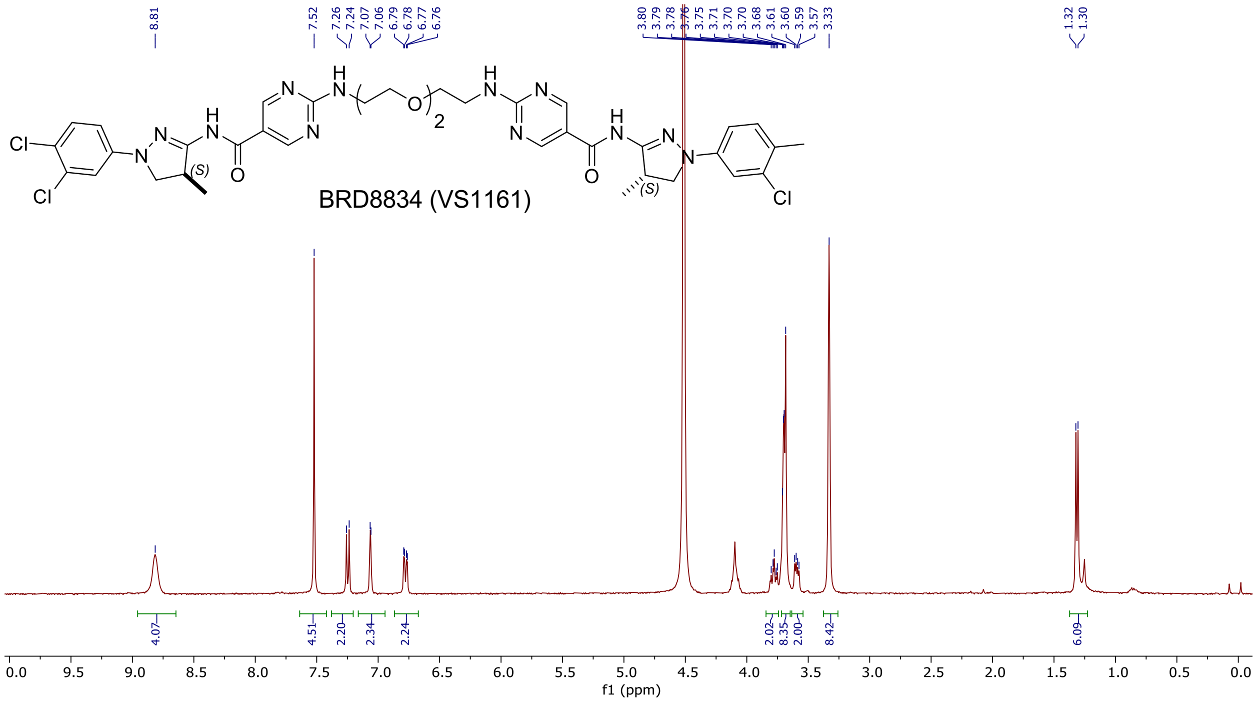
8.81
7.52
7.26
7.24
7.07
7.06
6.79
6.78
6.77
6.76
3.80
3.79
3.78
3.76
3.75
3.71
3.70
3.70
3.68
3.61
3.60
3.59
3.57
3.33
1.32
1.30
BRD8834 (VS1161)
4.07
4.51
2.20
2.34
2.24
2.02
8.35
2.00
8.42
6.09
f1 (ppm)

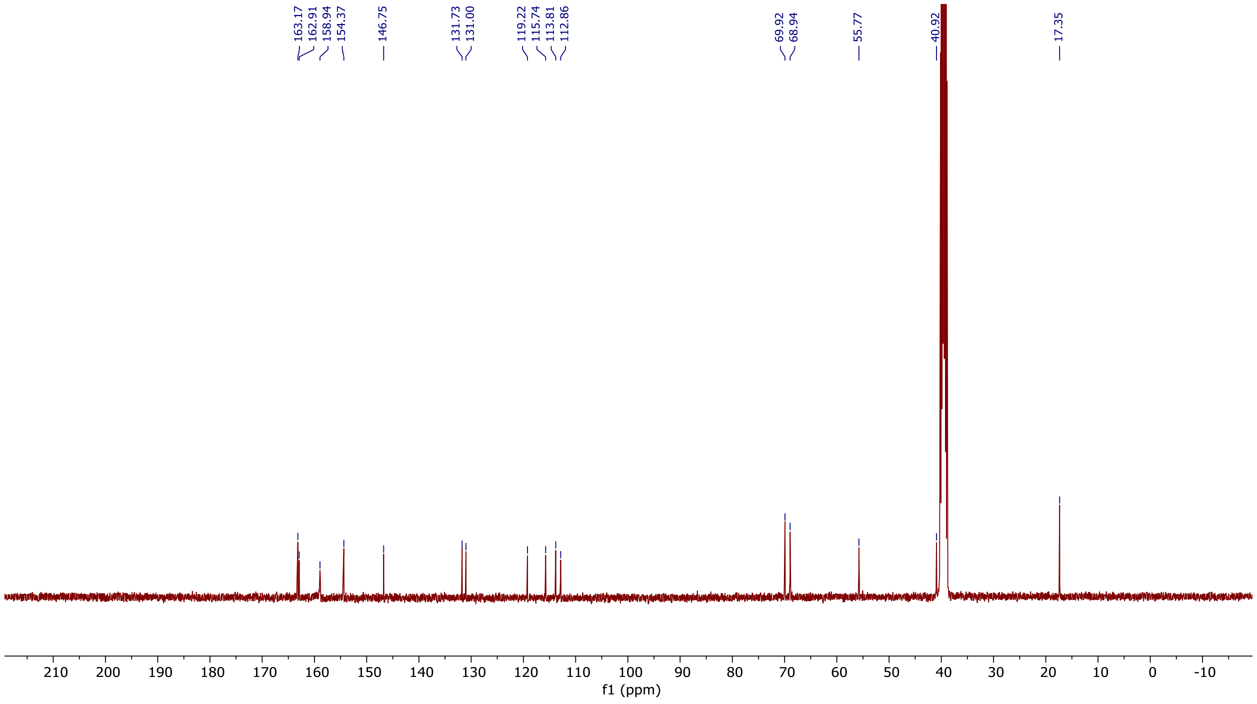
163.17
162.91
158.94
154.37
146.75
131.73
131.00
119.22
115.74
113.81
112.86
69.92
68.94
55.77
40.92
17.35
f1 (ppm)

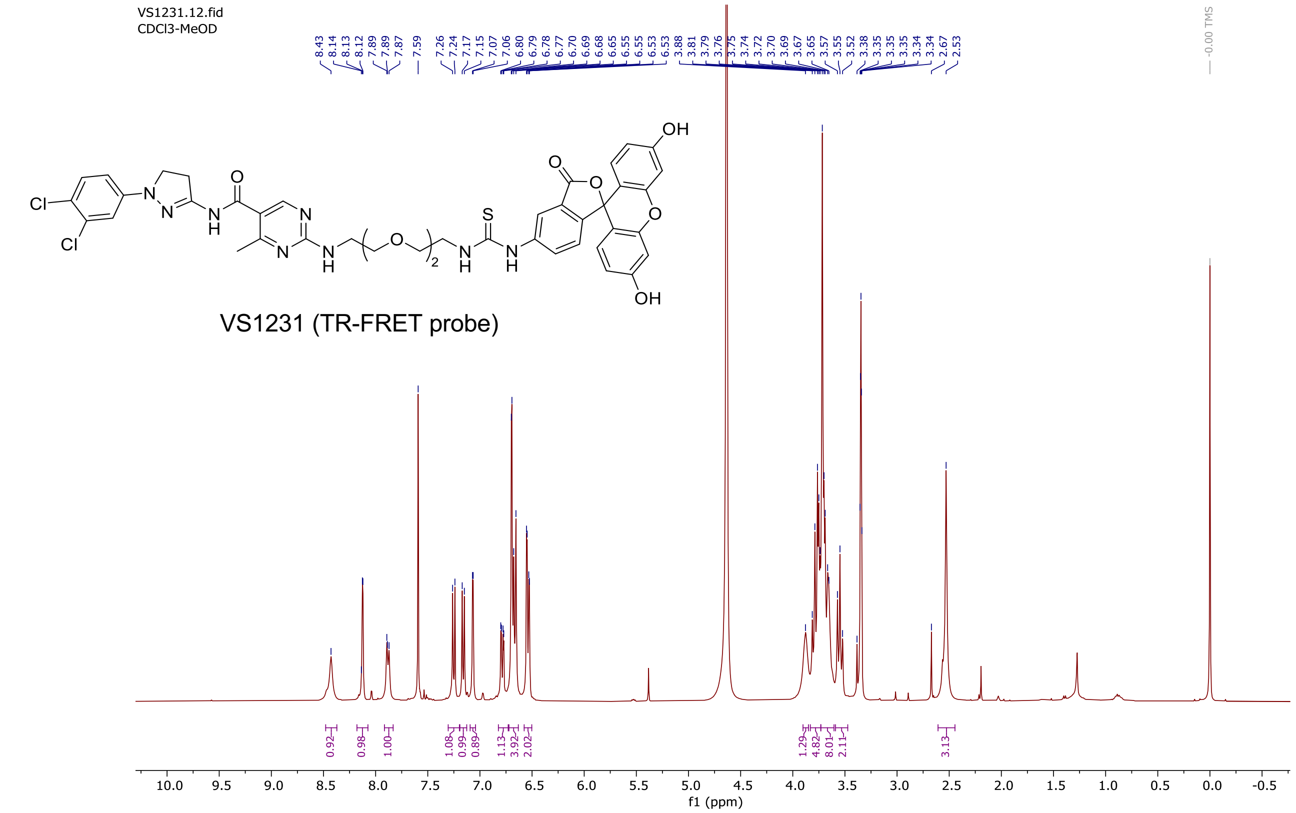
VS1231.12.fid
CDCl3-MeOD
8.43 8.14 8.13 8.12 7.89 7.89 7.87 7.59 7.26 7.24 7.17 7.15 7.07 7.06 6.80 6.79 6.78 6.77 6.70 6.69 6.68 6.65 6.55 6.55 6.53 6.53 3.88 3.81 3.79 3.76 3.75 3.74 3.72 3.70 3.69 3.67 3.65 3.57 3.55 3.52 3.38 3.35 3.35 3.35 3.34 3.34 2.67 2.53
-0.00 TMS
VS1231 (TR-FRET probe)
0.92 0.98 1.00 1.08 0.99 0.89 1.13 3.92 2.02 1.29 4.82 8.01 2.11 3.13
f1 (ppm)

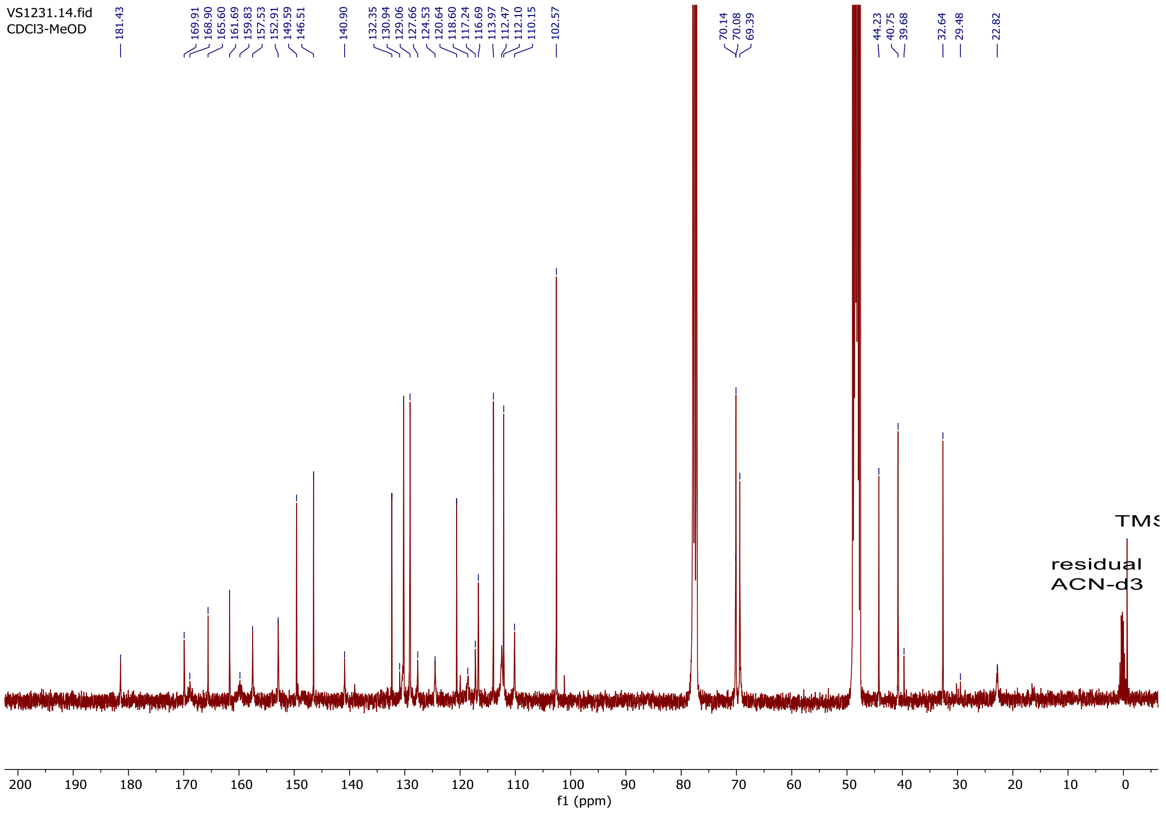
VS1231.14.fid
CDCl3-MeOD
181.43 169.91 168.90 165.60 161.69 159.83 157.53 152.91 149.59 146.51 140.90 132.35 130.94 129.06 127.66 124.53 120.64 118.60 117.24 116.69 113.97 112.47 112.10 110.15 102.57 70.14 70.08 69.39 44.23 40.75 39.68 32.64 29.48 22.82
TMS
residual
ACN-d3
f1 (ppm)

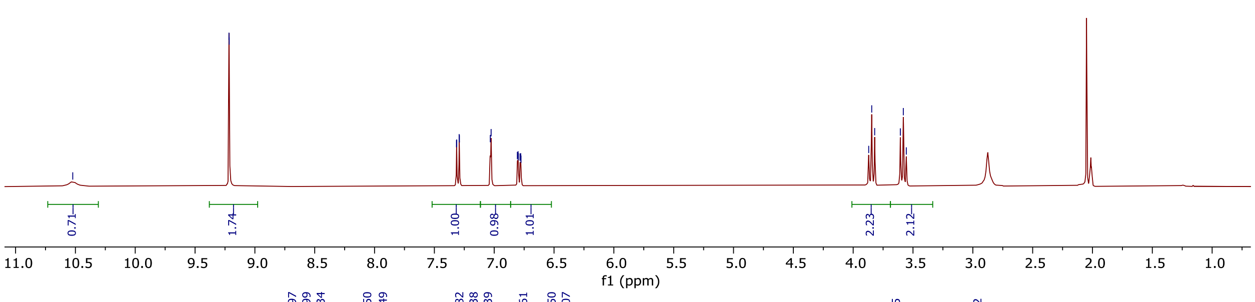
10.52
9.22
9.21
7.32
7.31
7.29
7.29
7.03
7.03
6.81
6.80
6.80
6.78
6.78
6.78
3.87
3.85
3.82
3.61
3.58
3.56
0.71
1.74
1.00
0.98
1.01
2.23
2.12
11.0 10.5 10.0 9.5 9.0 8.5 8.0 7.5 7.0 6.5 6.0 5.5 5.0 4.5 4.0 3.5 3.0 2.5 2.0 1.5 1.0
f1 (ppm)

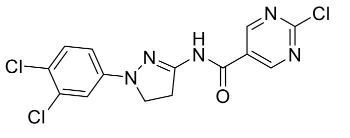
Cl
Cl
N
N
H
N
O
N
N
Cl

(VS1335)

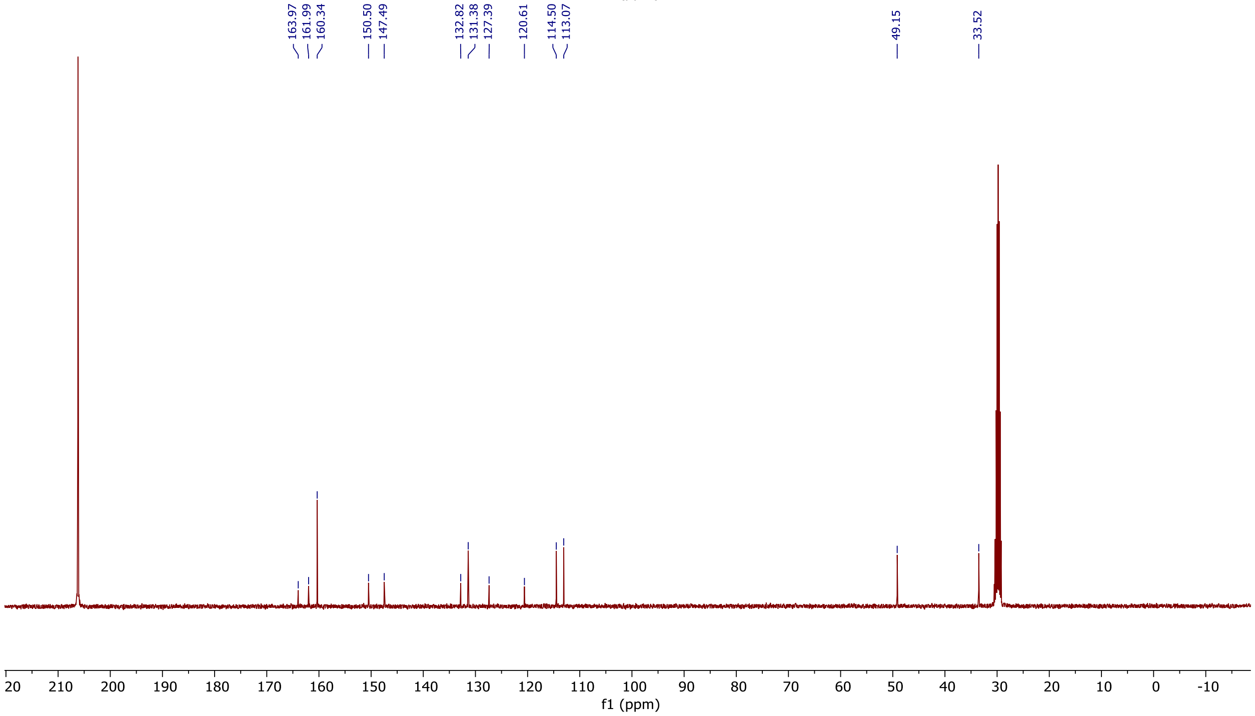
163.97
161.99
160.34
150.50
147.49
132.82
131.38
127.39
120.61
114.50
113.07
49.15
33.52
20 210 200 190 180 170 160 150 140 130 120 110 100 90 80 70 60 50 40 30 20 10 0 -10
f1 (ppm)

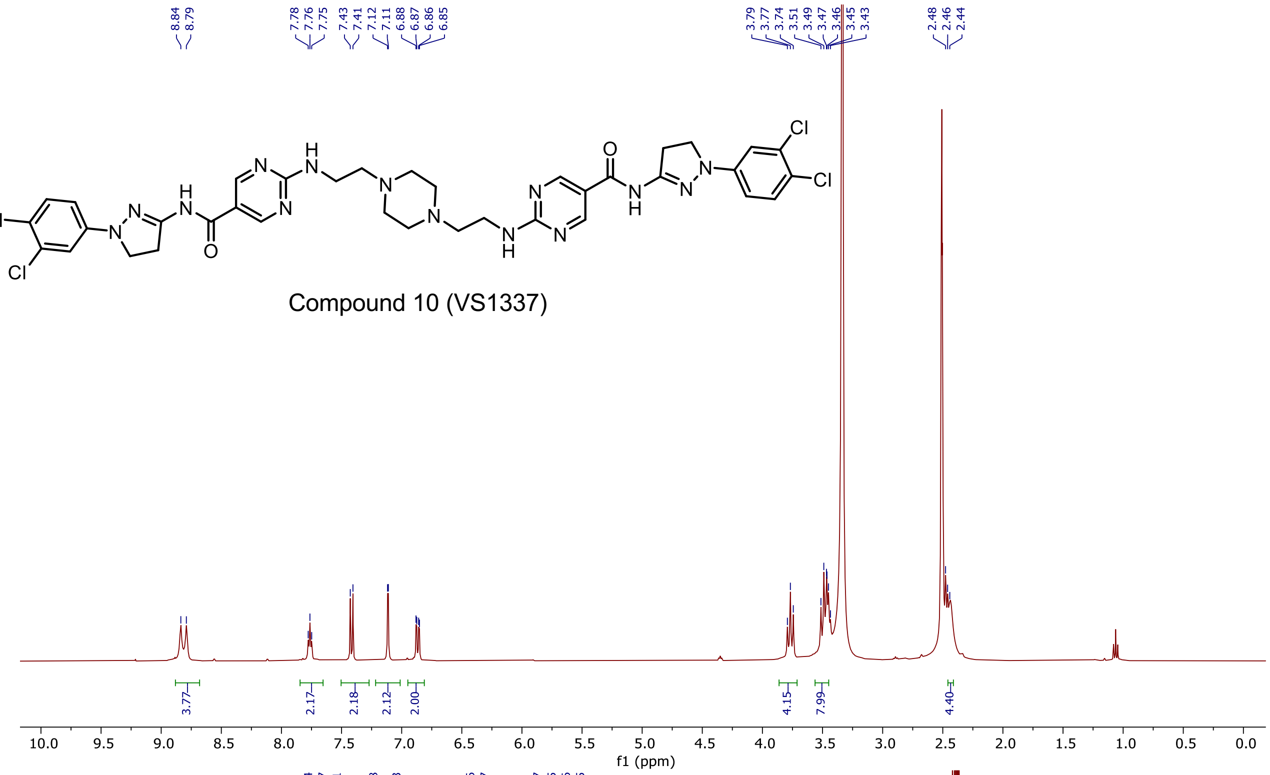
Compound 10 (VS1337)
8.84
8.79
7.78
7.76
7.75
7.43
7.41
7.12
7.11
6.88
6.87
6.86
6.85
3.79
3.77
3.74
3.51
3.49
3.47
3.46
3.45
3.43
2.48
2.46
2.44
3.77
2.17
2.18
2.12
2.00
4.15
7.99
4.40
f1 (ppm)

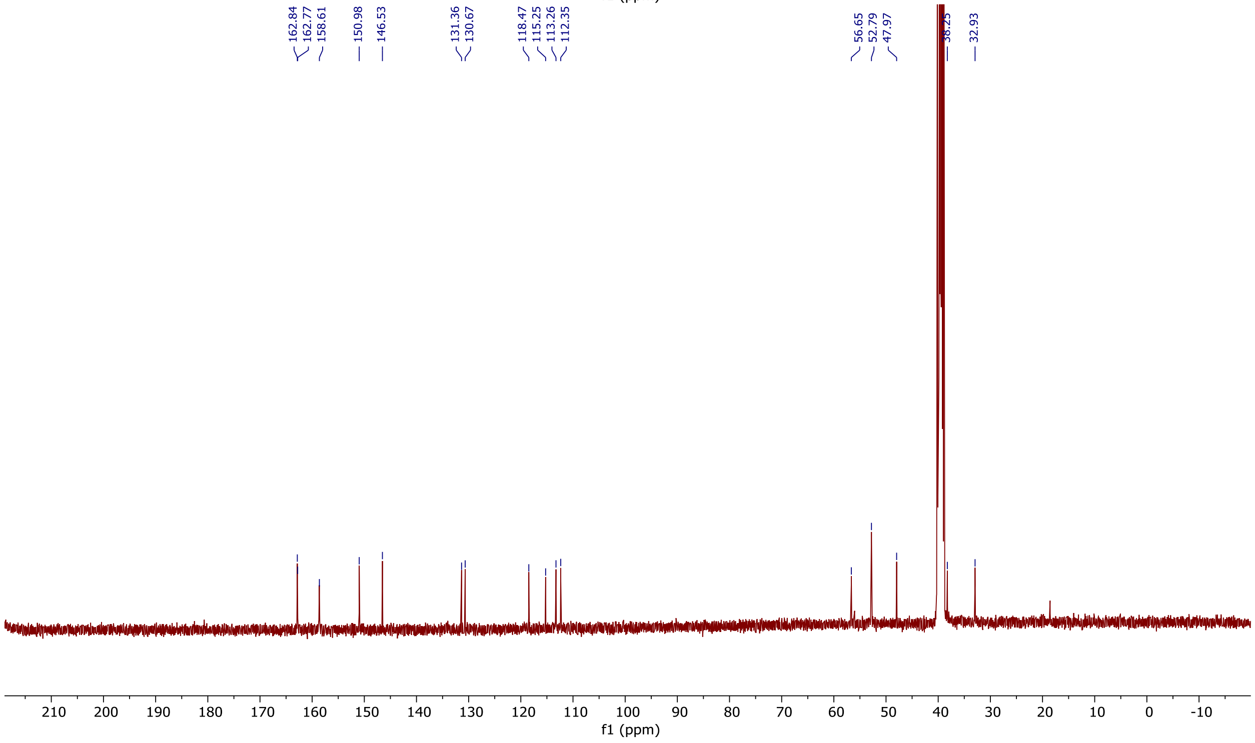
162.84
162.77
158.61
150.98
146.53
131.36
130.67
118.47
115.25
113.26
112.35
56.65
52.79
47.97
38.25
32.93
f1 (ppm)

2. SUPPORTING REFERENCES

(1) Pergu, R.; Shoba, V. M.; Chaudhary, S. K.; Munkanatta Godage, D. N. P.; Deb, A.; Singha, S.; Dhawa, U.; Singh, P.; Anokhina, V.; Singh, S.; et al. Development and Applications of Chimera Platforms for Tyrosine Phosphorylation. *ACS Central Science* **2023**, *9* (8), 1558-1566. DOI: 10.1021/acscentsci.3c00200.

(2) Shoba, V. M.; Munkanatta Godage, D. N. P.; Chaudhary, S. K.; Deb, A.; Siriwardena, S. U.; Choudhary, A. Synthetic Reprogramming of Kinases Expands Cellular Activities of Proteins. *Angew Chem Int Ed Engl* **2022**, *61* (29), e202202770. DOI: 10.1002/anie.202202770.

(3) Simpson, G. L.; Bertrand, S. M.; Borthwick, J. A.; Campobasso, N.; Chabanet, J.; Chen, S.; Coggins, J.; Cottom, J.; Christensen, S. B.; Dawson, H. C.; et al. Identification and Optimization of Novel Small c-Abl Kinase Activators Using Fragment and HTS Methodologies. *J Med Chem* **2019**, *62* (4), 2154-2171. DOI: 10.1021/acs.jmedchem.8b01872.
